# Overexpression of Transforming Acidic Coiled Coil‑Containing Protein 3 Reflects Malignant Characteristics and Poor Prognosis of Glioma

**DOI:** 10.3390/ijms18030235

**Published:** 2017-03-04

**Authors:** Ying Sun, Yu Tian, Guang-Zhi Wang, Shi-Hong Zhao, Bo Han, Yong-Li Li, Chuan-Lu Jiang

**Affiliations:** 1 Department of Neurosurgery, The 2nd Affiliated Hospital of Harbin Medical University, Harbin 150086, China; sunying701211@163.com (Y.S.); youihe001@163.com (Y.T.); wanggz_hmu@163.com (G.-Z.W.); zhaoshihong2013@163.com (S.-H.Z.); cable_han@163.com (B.H.); 2 Translational Medicine Research and Cooperation Center of Northern China, Heilongjiang Academy of Medical Science, Harbin 150086, China; 3 Sino-Russian Medical Research Center (SRMRC) Neuroscience Institute, Harbin 150086, China

**Keywords:** glioma, TACC3, biological process, glioma stem cells

## Abstract

Gliomas are malignant primary brain tumors with poor prognosis. Recently, research was indicative of a tight connection between tumor malignancy and genetic alterations. Here, we propose an oncogenic implication of transforming acidic coiled-coil-containing protein 3 (TACC3) in gliomas. By comprehensively analyzing the Chinese glioma genome atlas (CGGA) and publicly available data, we demonstrated that TACC3 were overexpressed along with glioma grade and served as an independent negative prognostic biomarker for glioma patients. Functions’ annotations and gene sets’ enrichment analysis suggested that TACC3 may participate in cell cycle, DNA repair, epithelium-mesenchymal transition and other tumor-related biological processes and molecular pathways. Patients with high TACC3 expression showed CD133^+^ stem cell properties, glioma plasticity and shorter overall survival time under chemo-/radio-therapy. Additionally, a TACC3 associated the miRNA-mRNA network was constructed based on in silico prediction and expression pattern, which provide a foundation for further detection of TACC3-miRNA-mRNA axis function. Collectively, our observations identify TACC3 as an oncogene of tumor malignancy, as well as a prognostic and motoring biomarker for glioma patients.

## 1. Introduction

Glioma is the most common and devastating primary brain tumor characterized by high infiltration, rapid progression and relative resistance to radiotherapy and most chemotherapeutic agents [1,2,3]. Although comprehensive multimodal treatments have been adopted, the outcomes for glioma patients remain poor. According to Chinese glioma genome atlas (CGGA) statistics, the overall survival (OS) times for glioblastoma (GBM, World Health Organization (WHO) Grade IV) patients is only 14.4 months, while the 6-month, 1-, 3- and 5-year OS rates were 87%, 61%, 15% and 9%, respectively [4]. With the evolution of biomedical techniques, especially the development of multidimensional omics platforms, some neuropathological biomarkers and molecular classification of glioma have been established. However, the identification of efficient diagnostic, prognostic and therapeutic biomarker and therapy targets remains crucial for glioma-tailored medicine.

The transforming acidic coiled-coil-containing protein 3 (TACC3) is a spindle-regulatory protein with a conserved C-terminal TACC domain, which is essential for its alignment with tubulin and promotes efficient microtubule elongation during mitosis [5,6]. Mounting evidence suggests that TACC3 dysregulation may function as an oncogene to induce multipolar spindle formation, cell cycle arrest, cell death and epithelium-mesenchymal transition (EMT) in certain types of tumors, including cervical cancer, gastric cancer, osteosarcoma, ovarian cancer, breast cancer and esophageal squamous cell carcinoma [7,8,9,10,11,12]. Moreover, whole-genome and RNA sequencing detect a recurrent fusion involving fibroblast growth factor receptor and TACC3 fusion (FGFR3-TACC3), which generates an oncogenic protein that promotes tumorigenesis and progression in various cancers [13,14,15]. These findings highlight the prognostic and therapeutic value for TACC3. However, the expression pattern and latent functions of TACC3 in glioma is not well investigated.

In present study, we analyzed CGGA sequencing and array data to explore the diagnostic, prognostic and therapeutic implications of TACC3 and delineate the TACC3-miRNA-mRNA network in glioma patients. We found that high expression of TACC3 was associated with WHO grade, expression subtype, isocitrate dehydrogenase (IDH) mutation and worse outcomes. Gene ontology and gene set enrichment analysis revealed that upregulated TACC3 expression was significantly correlated with cell cycle and DNA repair. Based on these observations, we identified that TACC3 could serve as a predictive marker for chemo- and radio-therapy. In addition, a TACC3-miRNA-mRNA network was constructed using miRWalk online analysis and CGGA miRNA data, which provided a foundation for further TACC3 function exploration.

## 2. Results

### 2.1. High Expression of Transforming Acidic Coiled-Coil-Containing Protein 3 (TACC3) Strongly Correlates with Glioma Grade

**Figure 1:**
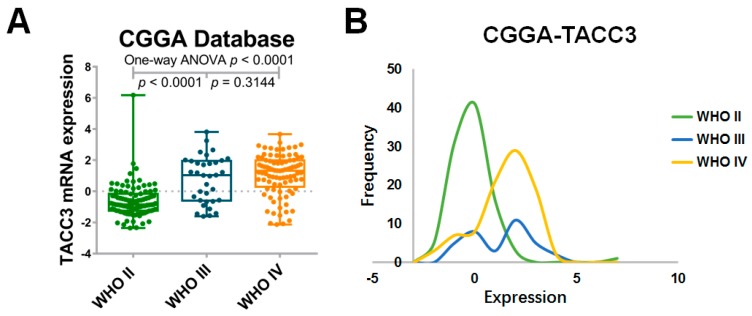

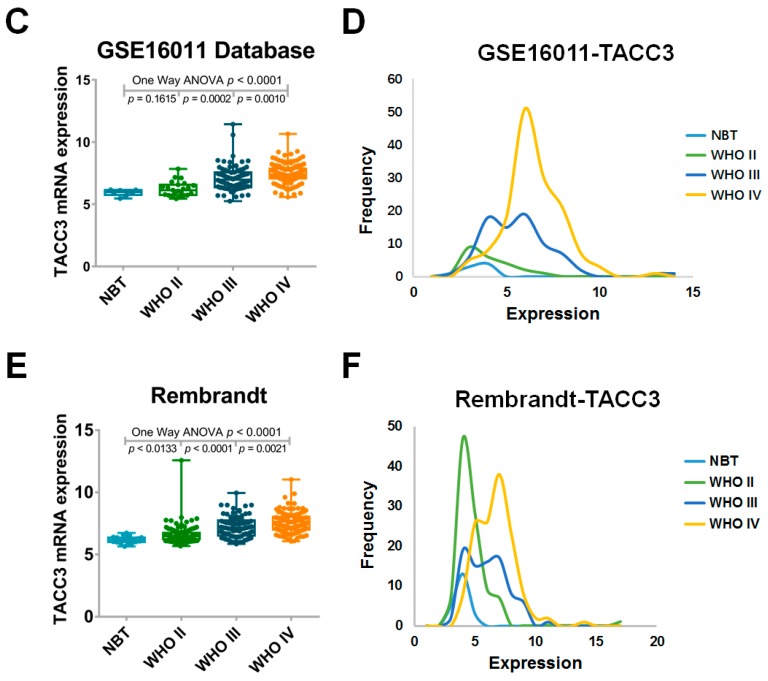
The expression pattern of transforming acidic coiled-coil-containing protein 3 (TACC3) in Chinese glioma genome atlas (CGGA), Gene Expression Omnibus (GSE) 16011 and Rembrandt database. (**A**) The expression of TACC3 is upregulated along with World Health Organization (WHO) grade (one-way ANOVA, *p* < 0.0001); (**B**) the distribution histogram of TACC3 in the CGGA cohort; (**C**)(**E**) GSE16011 and Rembrandt revealed the consistent consequences (one-way ANOVA, *p* < 0.0001, respectively); (**D**,**F**) the distribution histogram of GSE16011 and Rembrandt indicated that malignant gliomas presented with high TACC3 expression.

To explore the expression pattern of TACC3 in gliomas, mRNA expression microarray data and whole-genome sequencing data from independent datasets were obtained and analyzed. In the CGGA microarray cohort, the distribution of TACC3 was significantly different and overexpressed from Grades II–IV (Figure 1A,B, *p* < 0.0001). Consistently, analyses of Gene Expression Omnibus 16011 (GSE16011) and Rembrandt were indicative of a positive association between TACC3 expression and histological grades (Figure 1C–F, *p* < 0.0001, respectively). Besides, RNA sequencing data from CGGA and TCGA also showed that TACC3 was upregulated along with glioma grade, which further confirms the enrichment of TACC3 in glioma malignancy (Figure S1A, *p* < 0.0001; Figure S1B, *p* < 0.0001).

### 2.2. TACC3 as an Independent Prognostic Marker in Gliomas

**Figure 2:**
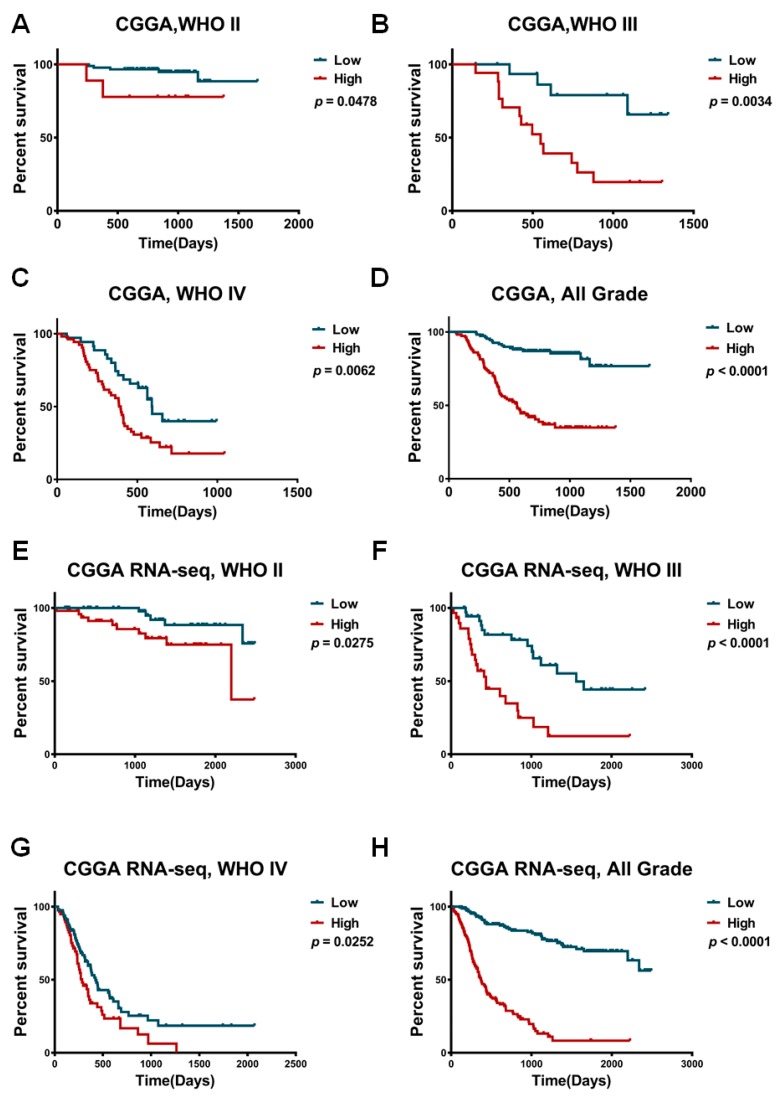
The prognostic implication of TACC3 in CGGA database. (**A**)–(**D**) Patients with high TACC3 expression presented with shorter overall survival time than those of low TACC3 expression in WHO II, III, IV and all grade glioma from the CGGA array cohort; (**E**)–(**H**) in the CGGA RNAseq cohort, TACC3 could stratify overall survival time significantly.

The high expression of TACC3 in malignant gliomas suggested that TACC3 may serve as an oncogene during glioma progression. Therefore, we speculated that TACC3 could stratify glioma patients according to its expression. To investigate the predictive implication of TACC3 in glioma prognosis, we analyzed TACC3 expression and the OS in the CGGA database. After eliminating patients with OS < 30 days, remaining patients went through Kaplan–Meier analysis. Glioma patients with high or low TACC3 expression had considerably different prognoses (Figure 2A, *p* = 0.0467; Figure 2B, *p* = 0.0034; Figure 2C, *p* = 0.0062). Similarly, analyses of RNAseq data demonstrated that TACC3 high expressed glioma had a shorter median OS than low expressed gliomas (Figure 2E, *p* = 0.0275; Figure 2F, *p* < 0.0001; Figure 2G, *p* = 0.0252).

Univariate and multivariate Cox analyses were performed to further explore the prognosis value of TACC3. In total, 217 gliomas patients with available OS were included (Table 1). The univariate Cox regression revealed that isocitrate dehydrogenase 1 (IDH1, hazards ratio (HR) = 0.244, *p* < 0.001), WHO grade (HR = 0.068, *p* < 0.001), TACC3 (HR = 0.169, *p* < 0.001), age (HR = 0.526, *p* = 0.004), Karnofsky performance score (KPS) (HR = 0.194, *p* < 0.001) were prognostic indicators for glioma patients. The multivariate Cox proportional hazards model identified TACC3 expression as an independent prognostic factor for OS (HR = 0.402, 95% confidence interval (CI) = 0.212–0.763, *p* = 0.005).

### 2.3. Functional Annotation of TACC3 Positive Correlated Genes

**Figure 3:**
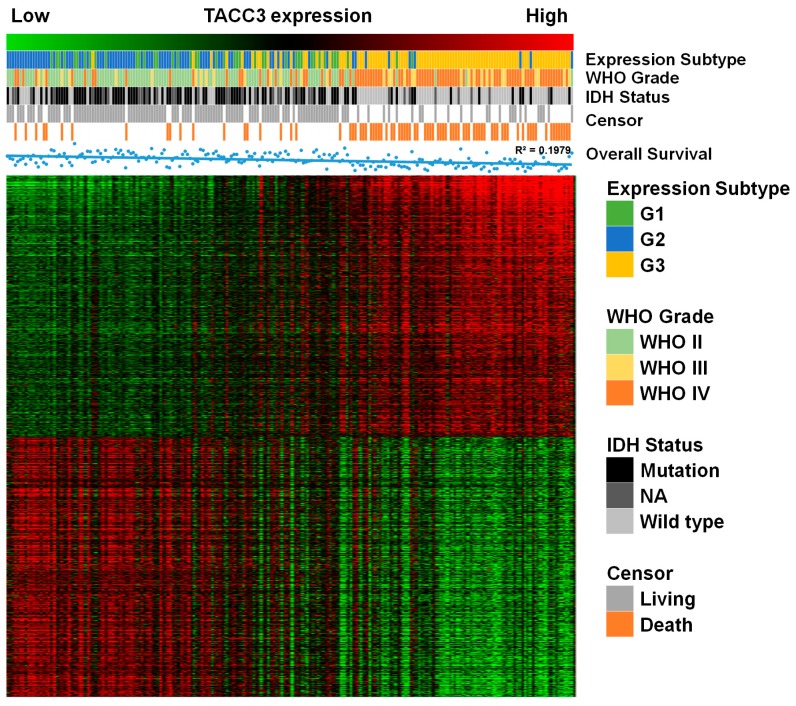
The TACC3-correlated genes in the CGGA array data. In pace with TACC3 upregulation, gliomas showed a lower rate of IDH mutation, shorter overall survival time, higher mortality and malignancy (G3 subtype and higher WHO grade). NA, not available.

**Figure 4:**
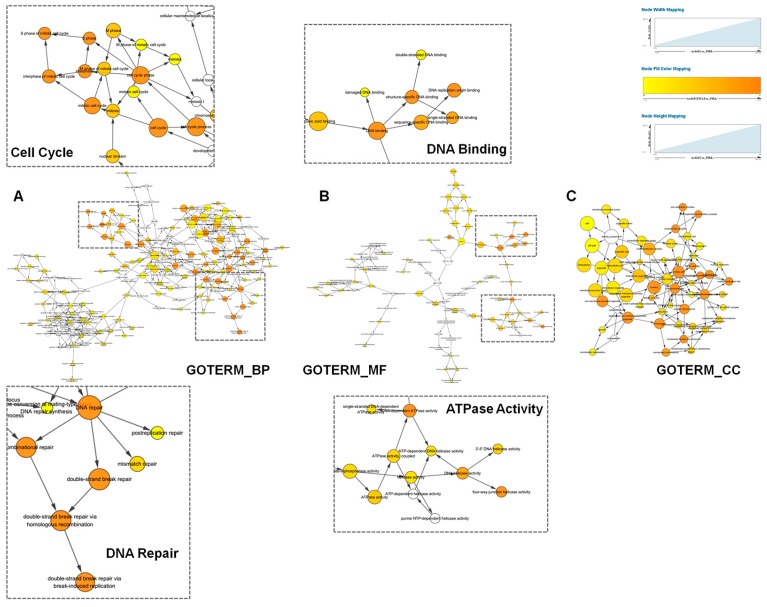
BiNGO annotation of TACC3 positive-related genes. (**A**) The biological process (BP) significantly was enriched in cell cycle and DNA repair-associated GO terms, while molecular functions (MF) were mainly gathered in ATPase activity and DNA binding. (**C**) The cellar component (CC) of TACC3 positive-related genes.

To further explore the functions of TACC3, we screened TACC3 positively- and negatively-associated genes using the Pearson correlation algorithm in the CGGA microarray dataset. The candidate genes were identified as *p* < 0.05 and *r* > 0.5 (or *r* < −0.5). In total, 679 positive and 654 negative genes were selected. A heat map of associated genes, the corresponding molecular biomarker and clinical parameters were constructed and presented as Figure 3. G3 subtype was characterized as the most malignant group of glioma with older age, lower IDH mutation and worse prognosis by the CGGA workgroup, while classical and mesenchymal are also regarded as glioma with poor outcomes. Our observations explicitly showed that along with TACC3 upregulation, patients were prone to be with the G3 subtype, classical/mesenchymal subtype and high grade glioma (Figure 3 and Table 2). Meanwhile, the OS was shorter and mortality was higher accompanied with TACC3 upregulation. Afterwards, TACC3 positively-associated genes were subject to GO analysis. As shown in Figure 4, BiNGO results demonstrated that the TACC3-associated biological processes were mainly enriched in cell cycle and DNA repair and molecular functions gathered in DNA binding and ATPase activity. Similarly, the results generated by the Database for Annotation, Visualization and Integrated Discovery (DAVID) also revealed that cell cycle-related GO terms (mitotic cell cycle, DNA replication, spindle organization, cell proliferation) and DNA repair-associated processes (double-strand break repair via homologous recombination, recombinational repair, mismatch repair) were significantly enriched (false discovery rate (FDR) < 0.05, Table S1). GSE16011 data were used as a validation (Figure S2). Consistently, TACC3 high expression was correlated with WHO grade, IDH status, OS and mortality. Moreover, cell cycle and DNA repair-associated GO biological processes were also significant for glioma patients with high TACC3 expression (Table S2).

### 2.4. Comparison of Different TACC3 Expression Groups by Gene Set Enrichment Analysis (GSEA)

**Figure 5:**
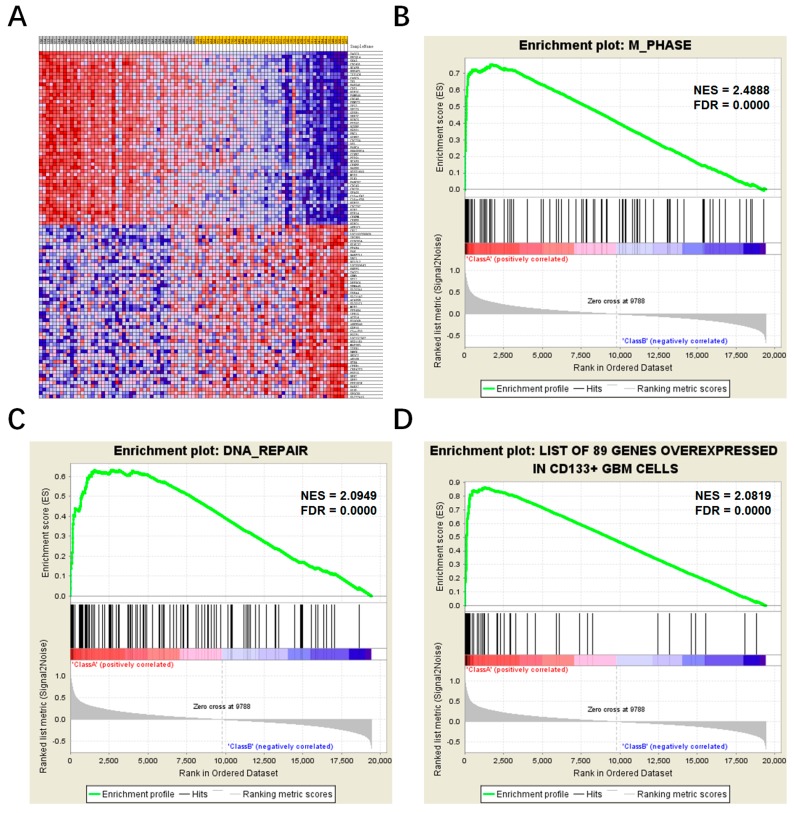
Gene Set Enrichment Analysis (GSEA) between TACC3 high and low expression subgroups in CGGA glioblastoma patients. (**A**) The heatmap of differential expressed genes between the two subgroups; (**B**,**C**) M phase (normalized enrichment score (NES) =2.0949, false discovery rate (FDR) = 0.0000) and DNA repair (NES = 2.0819, FDR = 0.0000) are significantly enriched in patients with high TACC3 expression; (**D**) a signature of CD133^+^-related genes also showed different enrichment in the TACC3 high expression group (NES = 2.0819, FDR = 0.0000).

To further confirm the TACC3-associated malignant behaviors and genetic alterations, we applied gene set enrichment analysis (GSEA) for GBM patients from CGGA microarray dataset. Eighty-nine GBM patients were divided into two subgroups according to TACC3 median expression. Enriched analyses of the GO biological process showed coincident results with annotations of TACC3-associated genes in gliomas (Figure 5). M phase, the mitotic procedure of the cell cycle, was the most significant process between the two groups (Figure 5B, normalized enrichment score (NES) = 2.4888, FDR = 0.0000). Meanwhile, the DNA repair process was meaningful in the comparison (Figure 5C, NES = 2.0948, FDR = 0.0000). DNA recombination, spindle, microtubule motor activity and replication fork were also the conspicuous GO gene sets enriched in the TACC3 high group (Table S3). Homologous recombination, base excision repair, cell cycle, DNA replication, mismatch repair, P53 signaling pathway and nucleotide excision repair were involved in TACC3-associated signaling pathways (Table S4). The enriched consequences of oncogenic signatures and hallmark gene sets were list in Tables S5 and S6, highlighting that overexpressed TACC3 were significantly associated with DNA repair processes, as well as cell proliferation-related functions and regulatory pathways.

Previous research demonstrated that glioma stem cells (GSCs) contribute to tumor maintenance, recurrence and therapeutics resistance [16]. The above results suggested that TACC3 may contributed to glioma proliferation and DNA repair. Thus, we supposed that TACC3 could serve as a surrogate GSC marker to predict the efficiency of clinical therapy. For this purpose, the signature of well-documented CD133^+^ GBM cells was quoted [17]. The GSEA results confirmed our hypothesis that TACC3 was significantly associated with GSCs’ corresponding signature (Figure 5D, NES =2.0819, FDR = 0.0000).

Moreover, the other collections of gene sets were analyzed with GSEA software to explore TACC3-related malignancy and molecular mechanisms. Notably, glioblastoma plasticity (Figure S3B) [18], EMT (Figure S3C) [19], tumorigenesis (Figure S3F) [20], tumor anaplastic (Figure S3D) [21], gliomagenesis by platelet derived growth factor B (PDGFB) (Figure S3A) [22], cancer metastasis signature (Figure S3E) [23], common cancer genes (Figure S3G) [24], targets of PTCH1 and SUFU (Figure S3H) and several other gene sets were remarkably enriched in the TACC3 high group, which confirmed the oncogenic role of TACC3 in tumor progression and partially uncovered the potential mechanism (Table S7).

### 2.5. TACC3 Could Serve as an Indicator for Therapy Efficiency

Our observations suggested that TACC3 was involved in the DNA damage response. Previous research has emphasized the association between DNA repair signaling pathway and chemo-/radio-resistance [25,26]. Therefore, we speculated that TACC3 could benefit tumor cells treated with therapeutic approaches, which will decrease the overall survival of glioma patients. To prove this hypothesis, we extracted patients with documented radiotherapy or chemotherapy in CGGA microarray datasets. Kaplan–Meier analysis revealed that patients with high TACC3 expression had a worse outcome treated with chemotherapy (Figure S4, *p* < 0.0001, HR = 0.284) or radiotherapy (Figure S4, *p* < 0.0001, HR = 0.155). Multivariable Cox analysis confirmed that TACC3 served as an independent prognostic indicator for glioma patients’ treatment (Table S8).

### 2.6. TACC3-miRNA-mRNA Network

**Figure 6:**
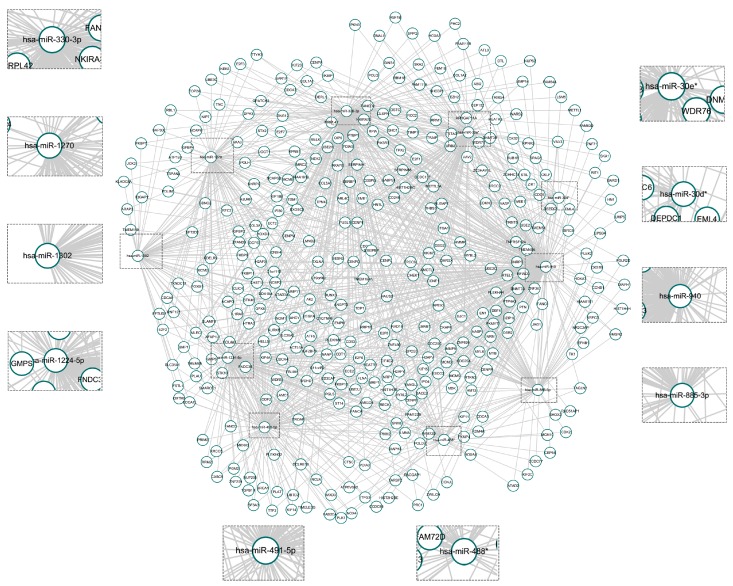
The TACC3-associated miRNA-mRNA network.

To identify TACC3-associated miRNAs, the miRWalk platform and CGGA miRNA/mRNA array data were utilized. A total of 10 miRNAs were negatively correlated with TACC3 expression and qualified as having a binding site within the TACC3 mRNA 3′ untraslational region. Some of these miRNAs, such as miR-1224-5p [27], miR-30e-3p [28], miR-488-3p [29], miR-491-5p [30], miR-940 [31] and miR-885-3p [32], are proven as tumor suppressors for various cancers. In the CGGA miRNA dataset, these TACC3 negatively associated miRNAs were significantly downregulated in high grade glioma (except for miR-330-3p, *p* = 0.4164) (Figure S5). By overlapping the miRNA targeted mRNAs with TACC3 positive-associated genes, a TACC3-miRNA-mRNA network was constructed with 380 mRNAs and 10 miRNAs (Figure 6).

## 3. Discussion

Although the dysfunction of TACC3 is thought to have oncogenic implications in a spectrum of cancers, little is known about its expression pattern and potent mechanism in glioma. In the present study, we analyzed transcriptome-clinical data from the CGGA dataset and revealed that TACC3 was upregulated along with glioma progression and inversely associated with patients’ prognosis. Functional annotations indicated that high TACC3 expression was tightly connected with tumor proliferation, DNA repair, stem properties, EMT and several other malignant biological processes. Our results further confirmed that TACC3 over-expression was indicative of a worse response to therapeutic approaches, suggesting a predictive value of TACC3 in clinical practice. Additionally, TACC3-related miRNA-mRNA interactions were identified after in silico prediction and large-scale verification, providing an alternative approach for miRNA-based glioma precision medicine.

Numerous investigations have emphasized the oncogenic effects of TACC3 in tumorigenesis and progression. Our findings suggested that this spindle-regulatory protein was involved in glioma progression. Patients with high TACC3 expression showed malignant clinical characteristics and biological processes. Regarding the regulatory effects of TACC3 in centrosome integrity, spindle stability and microtubule assembly during mitosis [8], it is rational that overexpressed TACC3 could lead to dysfunction of the mitotic procedure and result in sustained cell proliferation. TACC3-induced EMT empowered cancer cells with the abilities to invade, to resist apoptosis and to disseminate. This promotive effects could be partially attributed to the activation of the PI3K/Akt and ERK signaling pathways [9]. Meanwhile, TACC3 participated in the repair of DNA damage, protecting tumor cells from entering into apoptosis or other cell deaths. Thus, TACC3 could serve as a therapeutic target and hold great promise in clinical practice. Recent studies indicated that paclitaxel, a microtubule-interfering cancer drug, diminished TACC3 transcription in a time- and dose-dependent manner [33], providing an alternative modality for TACC3 intervention. However, considering TACC3 function in cell division, the usage of drugs should be cautious, and a full understanding TACC3 functions was crucial for developing precision therapeutic strategies [6].

Glioblastoma stem cells (GSCs) are regarded as one of the principle culprits for glioma progression and recurrence. Our observations suggested that patients with high TACC3 expression possessed the CD133^+^ property. To explain this phenomenon, we analyzed a collection of curated gene sets from the Molecular Signatures Database (MSigDB). Results indicated that Hedgehog (GCNP SHH UP LATE.V1 UP, GCNP SHH UP EARLY.V1 UP) [34] and Hippo (Cordenonsi YAP conserved signature) [35] associated signatures were significantly enriched in high TACC3 groups. Mounting evidence has proven that the Hedgehog-GLI and Hippo-YAP signaling pathways are indispensable for cancer cell malignant behaviors, especially for the maintenance of GSCs’ stem properties [36,37]. Notably, both of these two pathways were closely related with microtubule functions. Mammalian Hedgehog signal transduction requires a microtubule-based organelle, where glioma-associated oncogene homologs (GLIs) activity can be correctly regulated [38]. Besides, the MST1/2-SAV1 complex of the Hippo pathway promotes ciliogenesis and establishes a polarized cell structure in addition to regulating proliferation [39]. Moreover, MST1/2 could bind and phosphorylate Aurora kinase A (AURKA), while TACC3 is a well-characterized substrate of AURKA. The latent mechanism of TACC3, the Hedgehog and the Hippo signaling pathway needs to be investigated in the future.

Previous research showed that aberrant miRNAs expression could function as tumor suppressors or oncogenes in various human carcinogenesis, which extend our insights into the mechanisms of non-coding RNA underlying tumorigenesis. The importance of miRNA also indicated that modulation of miRNA expression could serve as a novel therapeutic approach. In glioma, the miR-181 family is well-verified as inhibitory miRNAs targeting MGMT, BCL-2 and KPNA4 [1,2,3], while miR-21, an oncogenic miRNA, was found to accelerate glioma progression and could be suppressed by small molecular drugs with high efficiency [40]; whereas, the miRNAs with TACC3 mRNA 3′ untranslational region binding capability have not been investigated. In the present study, we identified ten TACC3 negative-associated miRNAs in glioma patients. These miRNAs, downregulated along with WHO grade, showed potential in glioma miRNA-based therapy development. The accurate effects and molecular functions of these miRNAs need to be confirmed by experimental proof.

## 4. Materials and Methods

### 4.1. Microarray and RNA-Sequencing Data

Whole genome mRNA expression microarray data, RNA sequencing (RNAseq) data and relevant clinical characteristics (age, gender, Karnofsky performance score (KPS), WHO grade, OS, censor and pathological molecular biomarker) were downloaded from CGGA (http://www.cgga.org.cn) [41] and summarized in Table 1. CGGA represented a landmark to glioma research in China, which provides massive amounts of data for research, both in basic and clinical research of gliomas. Gene expression of CGGA RNAseq was calculated using the reads per kilobase transcriptome per million reads (RPKM) method, which can be directly used for gene comparison [42,43]. The Repository for Molecular Brain Neoplasia Data (REMBRANDT, http://cabig.cancer.gov/solutions/conductresearch/rembrandt), GSE16011 data (http://www.ncbi.nlm.nih.gov/geo/query/acc.cgi?acc=GSE16011) and the Cancer Genome Atlas (TCGA) RNAseq data (http://cancergenome.nih.gov/) were obtained as validation. CGGA microarray data were obtained for TACC3-associated miRNA selection. Prediction analysis of microarray (PAM) was performed to annotate CGGA expression data [44], according to the TCGA and CGGA subtype [41,45].

### 4.2. Functional Annotation and Gene Set Enrichment Analysis

Gene set enrichment analysis (GSEA) was performed using GSEA v2 software (www.broadinstitute.org/gsea). Annotated gene sets were downloaded from Molecular Signatures Database v5.1 (MSigDB, http://www.broad.mit.edu/gsea/msigdb/). Gene ontology (GO) analysis was performed using the online Database for Annotation, Visualization and Integrated Discovery (DAVID, http://david.ncifcrf.gov/) [46] and BiNGO (a plugin for Cytoscape, Software version 3.0.3) [47].

### 4.3. Integrative Analysis of TACC3-miRNA-mRNA Interactions

A total of 159 samples with mRNA microarray and miRNA expression data was analyzed. miRWalk 2.0 (http://zmf.umm.uni-heidelberg.de/apps/zmf/mirwalk2) is a comprehensive archive of predicted and validated miRNA-target interactions [48]. Various miRNA-target prediction datasets are documented to upgrade the comparative platform of miRNA binding sites. In the present study, 9 prediction programs (miRWalk, Microt4, miRanda, miRDB, miRMap, PITA, RNA22, RNAhybrid, Targetscan) were selected. miRNAs predicted by more than 3 platforms and negatively correlated with TACC3 were identified as candidates. Then, the TACC3 associated miRNAs were subjected to miRWalk 2.0 for its targeted mRNA screening. The miRNA negatively associated mRNAs with more than 3 algorithms predicted were identified. Collectively, the TACC3-miRNA-mRNA network was delineated using Cytoscape software (Softer version 3.3.0).

### 4.4. Statistical Analysis

The significant differences between two groups were estimated with the Student *t*-test. The *χ*^2^ test and Fisher’s exact test were used to compare the frequencies between groups. The difference of TACC3 among grades was performed by one-way analysis of variance (ANOVA). OS curves were plotted according to the Kaplan–Meier method, with the log-rank test applied for comparison. Pearson correlation analyses were conducted with R programming language (http://cran.r-project.org). Cox regression was used to determine prognostic value of TACC3 with OS in glioma patients. All differences were considered statistically significant at the level of *p* < 0.05. Statistics were performed using the SPSS 13.0 statistical software and GraphPad Prism 6.0.

## 5. Conclusions

In conclusion, our present work demonstrated that TACC3 is an oncogene for glioma progression. Patients with upregulated TACC3 expression showed malignant biological processes and worse outcomes. The regulatory network of TACC3 and associated miRNA-mRNA could serve as targets for molecular-based intervention. By comprehensively analyzing the transcriptome, miRNA data, the pathological biomarker and clinical features, our findings suggest that TACC3 expression could reflect malignant behaviors and poor outcomes, which are indicative of a potential prognostic and therapeutic implications for TACC3 in glioma management.

## Figures and Tables

**Table 1:** Variables related to OS in 217 gliomas: univariate and multivariate analysis.

Variable	Univariate		Multivariate	
	HR (95% CI)	*p*	HR (95% CI)	*p*
Gender				
Male	1.059 (0.683–1.642)	0.799		
Female				
Age (years)				
<45	0.526 (0.339–0.814)	0.004	0.795 (0.499–1.266)	0.333
≥45				
WHO Grade				
LGG	0.068 (0.031–0.149)	<0.001	0.170 (0.069–0.419)	<0.001
HGG				
KPS				
≥80	0.194 (0.125–0.304)	<0.001	0.279 (0.171–0.457)	<0.001
<80				
IDH1 status				
Mutation	0.244 (0.143–0.416)	<0.001	0.799 (0.437–1.460)	0.465
Wild-type				
TACC3 expression				
Low	0.169 (0.099–0.290)	<0.001	0.402 (0.212–0.763)	0.005
High				

HR, hazards ratio; CI, confidence interval; KPS, Karnofsky performance status; LGG, low grade glioma; HGG, high grade glioma; IDH1, isocitrate dehydrogenase 1.

**Table 2:** Clinical features of patients with different expression of TACC3 in the CGGA array database.

Variable		TACC3-Low (*n* = 110)	TACC3-High (*n* = 110)	*p*-Value
Median age		38	45	
Age	≥45	36	56	<0.01
	<45	74	54	
Gender	Male	56	68	>0.05
	Female	54	42	
Preoperative KPS score	≥80	95	79	<0.01
	<80	15	31	
Grade	WHO II	79	18	<0.01
	WHO III	13	21	
	WHO IV	18	71	
IDH1 status	Mutation	60	26	<0.01
	Wild type	29	72	
	NA	21	12	
CGGA subtype	G1	29	13	<0.01
	G2	70	12	
	G3	11	95	
TCGA subtype	Classical	12	26	<0.01
	Mesenchymal	14	62	
	Neural	50	5	
	Proneural	34	17	

## Supplementary Materials

Supplementary materials can be found at www.mdpi.com/1422-0067/18/3/235/s1.

## Abbreviations

CGGA	Chinese Glioma Genome Atlas
TCGA	The Cancer Genome Atlas
GBM	Glioblastoma
OS	Overall survival
TACC3	Transforming acidic coiled coil‑containing protein 3
IDH	Isocitrate dehydrogenase
EMT	Epithelium-mesenchymal transition
HR	Hazards ratio
95% CI	95% confidence interval
KPS	Karnofsky performance status
NES	Normalized enrichment score
GSCs	Glioma stem cells
GSEA	Gene set enrichment analysis
FDR	False discovery rate
