# Supplementary material for: Overexpression of Transforming Acidic Coiled Coil‑Containing Protein 3 Reflects Malignant Characteristics and Poor Prognosis of Glioma
Source: Int J Mol Sci. 2017 Mar 4;18(3):235. doi: 10.3390/ijms18030235 (PMC5372487; doi:10.3390/ijms18030235)
Supplement: Supplementary file 1 [file ijms-18-00235-s001.pdf]

Supplementary Materials: Overexpression of Transforming Acidic Coiled Coil-Containing Protein 3 Reflects Malignant Characteristics and Poor Prognosis of Glioma

Ying Sun, Yu Tian, Guang-Zhi Wang, Shi-Hong Zhao, Bo Han, Yong-Li Li and Chuan-Lu Jiang

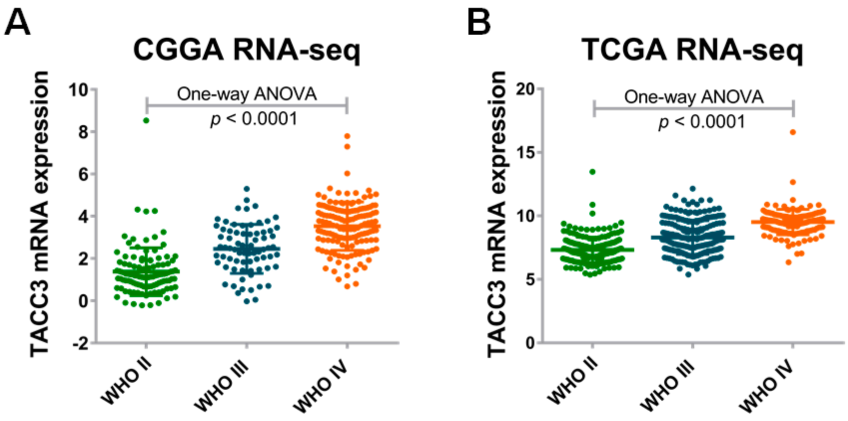

Figure S1. TACC3 expression in CGGA and TCGA RNA sequencing data. (**A**,**B**) TACC3 was upregulated along with WHO grade significantly (one-way ANOVA, $p < 0.0001$, respectively).

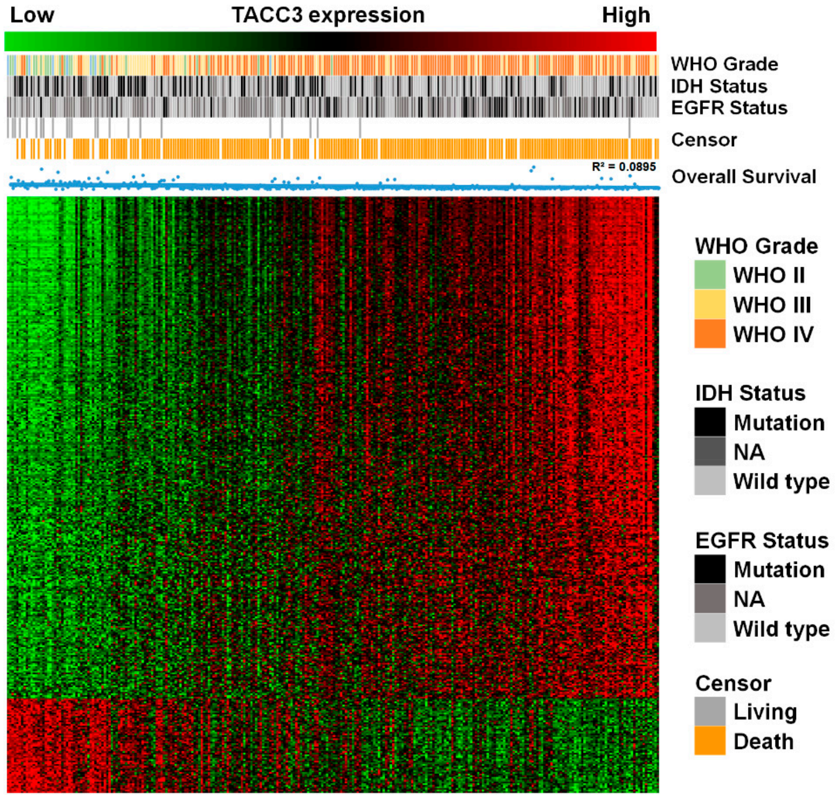

Figure S2. TACC3 associated-genes in GSE16011 datasets. WHO grade, mortality, IDH mutation frequency and overall survival were differently presented in pace with TACC3 expression.

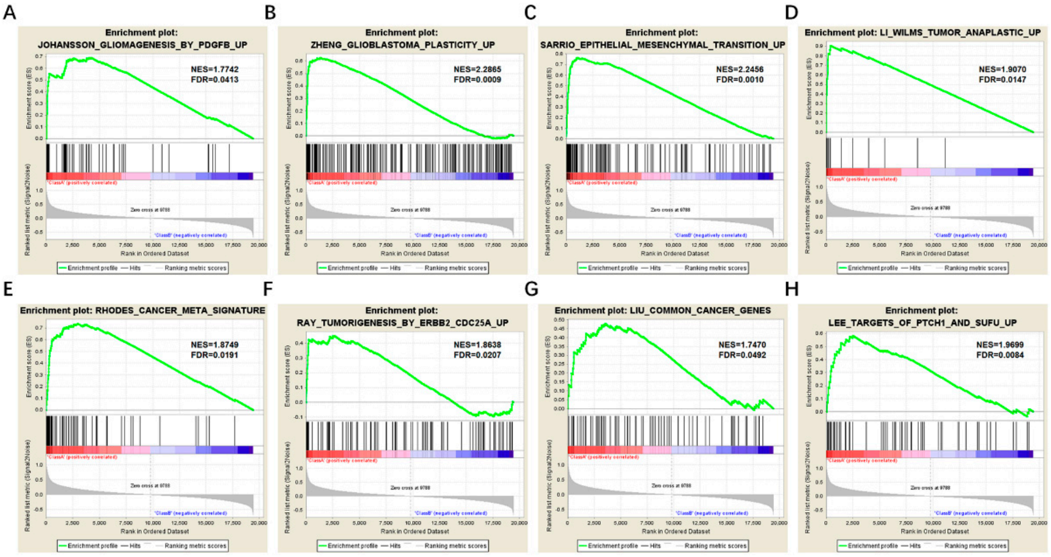

Figure S3. Gene Set Enrichment Analysis (GSEA) analyses of several gene sets were significantly enriched in the TACC3 high expression group.

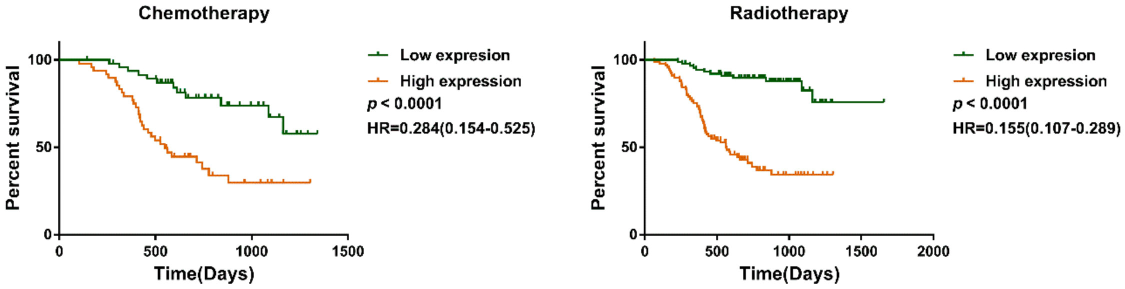

Figure S4. TACC3 could serve as an indicator for glioma patients with chemo- or radio-therapy.

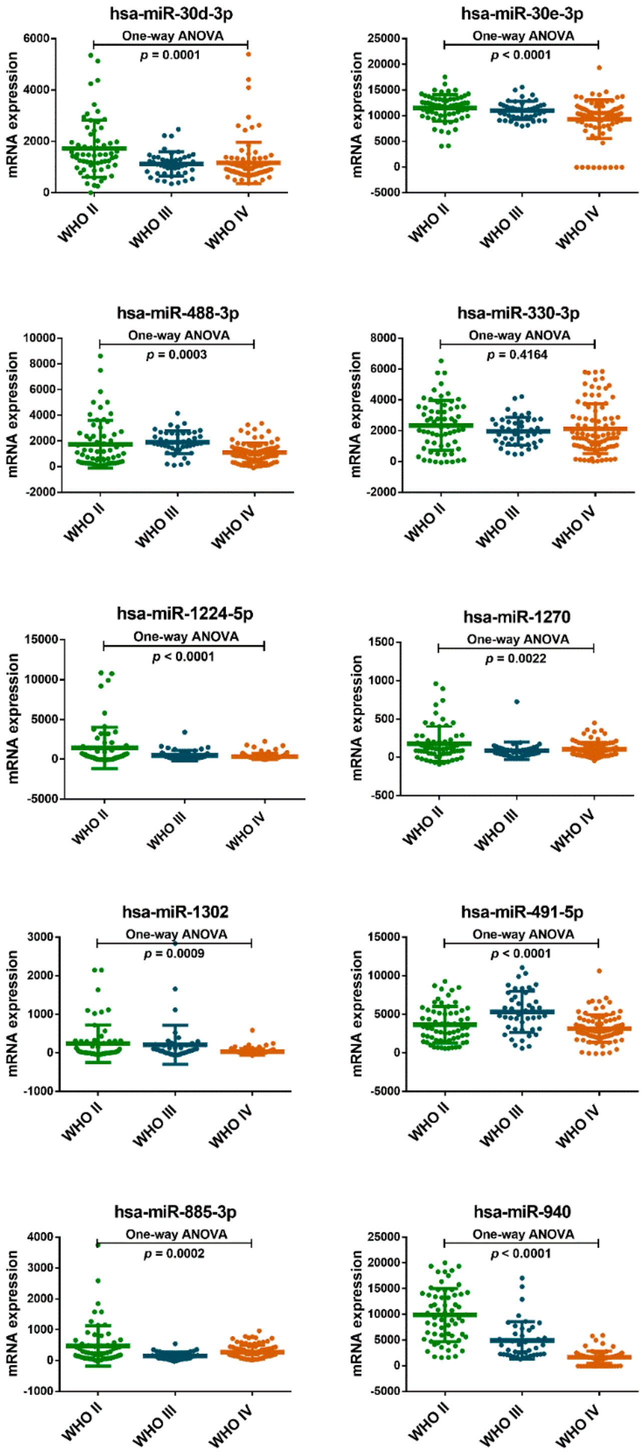

Figure S5. Expression of ten TACC3-related miRNAs in the CGGA dataset. miR-30d-3p, miR-30e-3p, miR-488-3p, miR-1224-5p, miR-1270, miR-1302, miR-491-5p, miR-885-3p and miR-940 were all significantly downregulated along with WHO grade (one-way ANOVA). miR-330-3p showed no difference.

Table S1. GO analyses for TACC3 positively-correlated genes (CGGA).

Category	Number	Term	Count	*p*	Fold Enrichment	FDR
BP_FAT	GO:0000070	mitotic sister chromatid segregation	15	2.03×10^{-11}	10.90264	3.03×10^{-55}
BP_FAT	GO:0000075	cell cycle checkpoint	22	1.99×10^{-11}	6.325929	5.81×10^{-55}
BP_FAT	GO:0000087	M phase of mitotic cell cycle	76	6.95×10^{-51}	8.877867	7.93×10^{-55}
BP_FAT	GO:0000278	mitotic cell cycle	93	5.79×10^{-50}	6.576946	2.64×10^{-48}
BP_FAT	GO:0000279	M phase	96	4.63×10^{-58}	7.635164	2.64×10^{-48}
BP_FAT	GO:0000280	nuclear division	76	1.54×10^{-51}	9.039283	1.19×10^{-47}
BP_FAT	GO:0006259	DNA metabolic process	91	3.16×10^{-36}	4.705805	5.42×10^{-33}
BP_FAT	GO:0006260	DNA replication	52	1.84×10^{-29}	7.161315	3.16×10^{-26}
BP_FAT	GO:0006261	DNA-dependent DNA replication	17	2.89×10^{-10}	7.669446	1.45×10^{-24}
BP_FAT	GO:0006270	DNA replication initiation	7	1.73×10^{-5}	11.44778	1.62×10^{-21}
BP_FAT	GO:0006310	DNA recombination	19	8.01×10^{-8}	4.734862	3.46×10^{-19}
BP_FAT	GO:0006323	DNA packaging	41	8.47×10^{-28}	9.169403	1.55×10^{-18}
BP_FAT	GO:0006325	chromatin organization	43	4.37×10^{-10}	2.976595	3.76×10^{-18}
BP_FAT	GO:0006333	chromatin assembly or disassembly	33	5.34×10^{-18}	6.799129	1.72×10^{-16}
BP_FAT	GO:0006334	nucleosome assembly	31	9.01×10^{-22}	9.656627	4.23×10^{-16}
BP_FAT	GO:0007017	microtubule-based process	36	3.34×10^{-11}	3.723274	9.16×10^{-15}
BP_FAT	GO:0007049	cell cycle	141	1.77×10^{-58}	4.754452	8.57×10^{-12}
BP_FAT	GO:0007051	spindle organization	17	3.71×10^{-12}	9.885063	1.07×10^{-9}
BP_FAT	GO:0007059	chromosome segregation	33	9.45×10^{-25}	10.66036	2.62×10^{-9}
BP_FAT	GO:0007067	mitosis	76	1.54×10^{-51}	9.039283	6.36×10^{-9}
BP_FAT	GO:0007076	mitotic chromosome condensation	7	4.08×10^{-6}	14.08957	8.37×10^{-9}
BP_FAT	GO:0007088	regulation of mitosis	12	7.01×10^{-6}	5.607074	3.42×10^{-8}
BP_FAT	GO:0007093	mitotic cell cycle checkpoint	12	4.24×10^{-7}	7.302236	3.49×10^{-8}
BP_FAT	GO:0007126	meiosis	20	4.22×10^{-9}	5.34007	5.42×10^{-8}
BP_FAT	GO:0007346	regulation of mitotic cell cycle	26	5.92×10^{-10}	4.475822	5.73×10^{-8}
BP_FAT	GO:0008283	cell proliferation	39	1.75×10^{-6}	2.340567	4.71×10^{-7}
BP_FAT	GO:0010564	regulation of cell cycle process	23	2.75×10^{-10}	5.279175	4.97×10^{-7}
BP_FAT	GO:0022402	cell cycle process	115	3.66×10^{-52}	5.325893	7.49×10^{-7}
BP_FAT	GO:0022403	cell cycle phase	106	3.39×10^{-58}	6.699595	1.02×10^{-6}
BP_FAT	GO:0030071	regulation of mitotic metaphase/anaphase transition	8	1.17×10^{-5}	9.515034	7.24×10^{-6}
BP_FAT	GO:0030261	chromosome condensation	9	2.59×10^{-6}	9.419884	7.24×10^{-6}
BP_FAT	GO:0031497	chromatin assembly	32	2.02×10^{-22}	9.624402	1.03×10^{-5}
BP_FAT	GO:0031577	spindle checkpoint	7	2.27×10^{-6}	15.2637	1.34×10^{-5}
BP_FAT	GO:0033554	cellular response to stress	59	4.88×10^{-12}	2.727587	1.72×10^{-5}
BP_FAT	GO:0034621	cellular macromolecular complex subunit organization	59	2.19×10^{-21}	4.32441	2.81×10^{-5}
BP_FAT	GO:0034622	cellular macromolecular complex assembly	52	1.15×10^{-18}	4.278773	1.07×10^{-4}
BP_FAT	GO:0034728	nucleosome organization	33	1.46×10^{-22}	9.284832	1.37×10^{-4}
BP_FAT	GO:0043933	macromolecular complex subunit organization	74	5.01×10^{-15}	2.727196	3.80×10^{-4}
BP_FAT	GO:0048015	phosphoinositide-mediated signaling	14	2.72×10^{-5}	4.162828	3.80×10^{-4}
BP_FAT	GO:0048285	organelle fission	76	4.36×10^{-50}	8.684027	7.28×10^{-4}
BP_FAT	GO:0051276	chromosome organization	71	1.33×10^{-22}	3.830537	0.003008
BP_FAT	GO:0051301	cell division	78	2.39×10^{-43}	6.918559	0.003898
BP_FAT	GO:0051321	meiotic cell cycle	20	6.00×10^{-9}	5.233269	0.004436
BP_FAT	GO:0051325	interphase	20	1.64×10^{-8}	4.937046	0.007007
BP_FAT	GO:0051327	M phase of meiotic cell cycle	20	4.22×10^{-9}	5.34007	0.012023
BP_FAT	GO:0051329	interphase of mitotic cell cycle	20	1.00×10^{-8}	5.080844	0.012023
BP_FAT	GO:0051726	regulation of cell cycle	44	1.53×10^{-12}	3.478306	0.020027
BP_FAT	GO:0051783	regulation of nuclear division	12	7.01×10^{-6}	5.607074	0.02964
BP_FAT	GO:0065003	macromolecular complex assembly	67	6.21×10^{-13}	2.636308	0.044333
BP_FAT	GO:0065004	protein-DNA complex assembly	34	4.47×10^{-24}	9.776436	0.046641
BP_FAT	GO:0000226	microtubule cytoskeleton organization	24	7.83×10^{-9}	4.272056	6.28×10^{-49}
BP_FAT	GO:0000724	double-strand break repair via homologous recombination	9	2.21×10^{-7}	12.39458	7.49×10^{-47}
BP_FAT	GO:0000725	recombinational repair	9	2.21×10^{-7}	12.39458	9.93×10^{-47}
BP_FAT	GO:0000819	sister chromatid segregation	15	3.16×10^{-11}	10.60798	4.10×10^{-40}
BP_FAT	GO:0006281	DNA repair	50	2.47×10^{-19}	4.606751	7.68×10^{-21}

BP_FAT	GO:0006297	nucleotide-excision repair, DNA gap filling	7	2.58×10^{-5}	10.77438	2.29×10^{-19}
BP_FAT	GO:0006302	double-strand break repair	15	6.24×10^{-8}	6.330567	2.50×10^{-19}
BP_FAT	GO:0006974	response to DNA damage stimulus	58	1.00×10^{-19}	4.068761	1.98×10^{-15}
CC_FAT	GO:0005694	chromosome	112	3.31×10^{-63}	6.870064	4.53×10^{-60}
CC_FAT	GO:0044427	chromosomal part	100	9.53×10^{-59}	7.309931	1.30×10^{-55}
CC_FAT	GO:0000793	condensed chromosome	46	4.40×10^{-33}	10.06164	6.03×10^{-30}
CC_FAT	GO:0000775	chromosome, centromeric region	45	9.82×10^{-33}	10.2398	1.34×10^{-29}
CC_FAT	GO:0043232	intracellular non-membrane-bounded organelle	198	1.98×10^{-30}	2.152093	2.71×10^{-27}
CC_FAT	GO:0043228	non-membrane-bounded organelle	198	1.98×10^{-30}	2.152093	2.71×10^{-27}
CC_FAT	GO:0000779	condensed chromosome, centromeric region	31	1.81×10^{-26}	13.25313	2.48×10^{-23}
CC_FAT	GO:0031981	nuclear lumen	133	3.38×10^{-26}	2.588119	4.62×10^{-23}
CC_FAT	GO:0000777	condensed chromosome kinetochore	29	1.06×10^{-25}	14.10817	1.45×10^{-22}
CC_FAT	GO:0005819	spindle	40	8.69×10^{-24}	7.677914	1.19×10^{-20}
CC_FAT	GO:0032993	protein-DNA complex	32	1.33×10^{-23}	10.4991	1.83×10^{-20}
CC_FAT	GO:0005654	nucleoplasm	95	4.75×10^{-23}	3.039174	6.50×10^{-20}
CC_FAT	GO:0000776	kinetochore	30	8.71×10^{-23}	10.99338	1.19×10^{-19}
CC_FAT	GO:0043233	organelle lumen	145	1.24×10^{-22}	2.248005	1.70×10^{-19}
CC_FAT	GO:0031974	membrane-enclosed lumen	146	2.86×10^{-22}	2.219604	3.92×10^{-19}
CC_FAT	GO:0070013	intracellular organelle lumen	142	3.56×10^{-22}	2.252231	4.88×10^{-19}
CC_FAT	GO:0000786	nucleosome	25	3.32×10^{-19}	11.19696	4.54×10^{-16}
CC_FAT	GO:0000785	chromatin	40	1.18×10^{-18}	5.643267	1.62×10^{-15}
CC_FAT	GO:0015630	microtubule cytoskeleton	61	3.48×10^{-15}	3.135148	4.71×10^{-12}
CC_FAT	GO:0000228	nuclear chromosome	29	2.54×10^{-12}	5.051072	3.48×10^{-9}
CC_FAT	GO:0005657	replication fork	12	6.31×10^{-9}	10.58113	8.64×10^{-6}
CC_FAT	GO:0044454	nuclear chromosome part	21	9.26×10^{-9}	4.85691	1.27×10^{-5}
CC_FAT	GO:0005876	spindle microtubule	11	3.03×10^{-8}	10.70275	4.15×10^{-5}
CC_FAT	GO:0044430	cytoskeletal part	67	8.19×10^{-8}	1.985814	1.12×10^{-4}
CC_FAT	GO:0005874	microtubule	30	1.33×10^{-7}	3.08938	1.82×10^{-4}
CC_FAT	GO:0000922	spindle pole	10	1.90×10^{-6}	8.298922	0.002607
CC_FAT	GO:0005856	cytoskeleton	82	2.65×10^{-6}	1.675409	0.003633
CC_FAT	GO:0005815	microtubule organizing center	26	3.47×10^{-6}	2.899702	0.004751
CC_FAT	GO:0000940	outer kinetochore of condensed chromosome	6	6.06×10^{-6}	18.81089	0.008294
CC_FAT	GO:0005813	centrosome	23	1.47×10^{-5}	2.897213	0.020161
MF_FAT	GO:0003677	DNA binding	139	1.02×10^{-11}	1.728103	1.50×10^{-8}
MF_FAT	GO:0005524	ATP binding	89	1.39×10^{-7}	1.746251	2.05×10^{-4}
MF_FAT	GO:0032559	adenyl ribonucleotide binding	89	2.52×10^{-7}	1.722921	3.71×10^{-4}
MF_FAT	GO:0000166	nucleotide binding	118	1.07×10^{-6}	1.52322	0.001581
MF_FAT	GO:0032555	purine ribonucleotide binding	101	1.21×10^{-6}	1.594211	0.001781
MF_FAT	GO:0032553	ribonucleotide binding	101	1.21×10^{-6}	1.594211	0.001781
MF_FAT	GO:0030554	adenyl nucleotide binding	90	1.26×10^{-6}	1.653895	0.001851
MF_FAT	GO:0003697	single-stranded DNA binding	12	2.17×10^{-6}	6.32289	0.003196
MF_FAT	GO:0001883	purine nucleoside binding	90	2.36×10^{-6}	1.629102	0.003479
MF_FAT	GO:0001882	nucleoside binding	90	3.17×10^{-6}	1.617985	0.004667
MF_FAT	GO:0017076	purine nucleotide binding	102	4.84×10^{-6}	1.541163	0.007127
MF_FAT	GO:0043566	structure-specific DNA binding	18	1.01×10^{-5}	3.597506	0.014834
MF_FAT	GO:0003777	microtubule motor activity	13	1.15×10^{-5}	4.892712	0.017
MF_FAT	GO:0003682	chromatin binding	18	1.58×10^{-5}	3.477589	0.023314
MF_FAT	GO:0008094	DNA-dependent ATPase activity	11	2.14×10^{-5}	5.592614	0.031522
MF_FAT	GO:0004386	helicase activity	17	2.52×10^{-5}	3.518989	0.037187
KEGG_PATHWAY	hsa04110	Cell cycle	36	1.63×10^{-20}	6.843364	1.85×10^{-17}
KEGG_PATHWAY	hsa03030	DNA replication	18	8.47×10^{-15}	11.88084	9.58×10^{-12}
KEGG_PATHWAY	hsa05322	Systemic lupus erythematosus	24	7.28×10^{-12}	5.760408	8.26×10^{-9}
KEGG_PATHWAY	hsa03440	Homologous recombination	10	1.16×10^{-6}	8.486315	0.001314
KEGG_PATHWAY	hsa00240	Pyrimidine metabolism	16	7.37×10^{-6}	4.001968	0.008365
KEGG_PATHWAY	hsa03430	Mismatch repair	8	2.83×10^{-5}	8.264933	0.032165

Table S2. GO analyses for TACC3 positively-correlated genes (GSE16011).

Category	Number	Term	Count	*p*	Fold Enrichment	FDR
BP_FAT	GO:0007049	cell cycle	126	4.36×10^{-87}	8.513786	7.00×10^{-84}
BP_FAT	GO:0022403	cell cycle phase	97	2.30×10^{-80}	12.28529	3.68×10^{-77}
BP_FAT	GO:0000279	M phase	89	4.21×10^{-79}	14.1843	6.75×10^{-76}
BP_FAT	GO:0022402	cell cycle process	106	5.69×10^{-78}	9.837196	9.13×10^{-75}
BP_FAT	GO:0000278	mitotic cell cycle	85	1.02×10^{-68}	12.04567	1.63×10^{-65}
BP_FAT	GO:0000087	M phase of mitotic cell cycle	70	6.53×10^{-66}	16.38566	1.05×10^{-62}
BP_FAT	GO:0000280	nuclear division	69	4.93×10^{-65}	16.44524	7.92×10^{-62}
BP_FAT	GO:0007067	mitosis	69	4.93×10^{-65}	16.44524	7.92×10^{-62}
BP_FAT	GO:0048285	organelle fission	69	1.11×10^{-63}	15.79892	1.78×10^{-60}
BP_FAT	GO:0051301	cell division	71	4.32×10^{-58}	12.61973	6.93×10^{-55}
BP_FAT	GO:0006259	DNA metabolic process	76	1.22×10^{-46}	7.875479	1.96×10^{-43}
BP_FAT	GO:0006260	DNA replication	45	1.18×10^{-35}	12.4186	1.89×10^{-32}
BP_FAT	GO:0007059	chromosome segregation	30	8.07×10^{-30}	19.42004	1.30×10^{-26}
BP_FAT	GO:0006974	response to DNA damage stimulus	50	1.41×10^{-27}	7.028701	2.27×10^{-24}
BP_FAT	GO:0006281	DNA repair	44	9.16×10^{-27}	8.123594	1.47×10^{-23}
BP_FAT	GO:0007017	microtubule-based process	40	1.31×10^{-24}	8.289978	2.10×10^{-21}
BP_FAT	GO:0051726	regulation of cell cycle	40	2.73×10^{-20}	6.336448	4.37×10^{-17}
BP_FAT	GO:0033554	cellular response to stress	50	1.52×10^{-19}	4.631988	2.44×10^{-16}
BP_FAT	GO:0000226	microtubule cytoskeleton organization	28	2.49×10^{-19}	9.987449	4.00×10^{-16}
BP_FAT	GO:0051276	chromosome organization	46	3.81×10^{-19}	4.973132	6.11×10^{-16}
BP_FAT	GO:0000070	mitotic sister chromatid segregation	17	9.19×10^{-19}	24.76055	1.47×10^{-15}
BP_FAT	GO:0000819	sister chromatid segregation	17	1.59×10^{-18}	24.09135	2.55×10^{-15}
BP_FAT	GO:0007051	spindle organization	18	2.15×10^{-18}	20.97364	3.44×10^{-15}
BP_FAT	GO:0000075	cell cycle checkpoint	21	3.30×10^{-16}	12.10018	5.33×10^{-13}
BP_FAT	GO:0051321	meiotic cell cycle	21	2.53×10^{-15}	11.01116	4.10×10^{-12}
BP_FAT	GO:0007126	meiosis	20	2.31×10^{-14}	10.70084	3.71×10^{-11}
BP_FAT	GO:0051327	M phase of meiotic cell cycle	20	2.31×10^{-14}	10.70084	3.71×10^{-11}
BP_FAT	GO:0007346	regulation of mitotic cell cycle	23	1.11×10^{-13}	7.934109	1.77×10^{-10}
BP_FAT	GO:0006323	DNA packaging	20	6.68×10^{-13}	8.963095	1.07×10^{-9}
BP_FAT	GO:0006261	DNA-dependent DNA replication	15	2.70×10^{-12}	13.56055	4.33×10^{-9}
BP_FAT	GO:0010564	regulation of cell cycle process	19	4.51×10^{-12}	8.739018	7.24×10^{-9}
BP_FAT	GO:0006297	nucleotide-excision repair, DNA gap filling	10	5.97×10^{-12}	30.84359	9.58×10^{-9}
BP_FAT	GO:0006310	DNA recombination	18	1.19×10^{-11}	8.988704	1.91×10^{-8}
BP_FAT	GO:0051329	interphase of mitotic cell cycle	17	9.28×10^{-11}	8.654173	1.49×10^{-7}
BP_FAT	GO:0051325	interphase	17	1.45×10^{-10}	8.409244	2.32×10^{-7}
BP_FAT	GO:0006289	nucleotide-excision repair	13	3.64×10^{-10}	12.39352	5.83×10^{-7}
BP_FAT	GO:0006302	double-strand break repair	13	1.59×10^{-9}	10.99425	2.55×10^{-6}
BP_FAT	GO:0007018	microtubule-based movement	16	3.47×10^{-9}	7.424299	5.56×10^{-6}
BP_FAT	GO:0007010	cytoskeleton organization	30	4.24×10^{-9}	3.607852	6.81×10^{-6}
BP_FAT	GO:0048015	phosphoinositide-mediated signaling	14	1.05×10^{-8}	8.34179	1.68×10^{-5}
BP_FAT	GO:0030261	chromosome condensation	9	1.24×10^{-8}	18.87628	2.00×10^{-5}
BP_FAT	GO:0007076	mitotic chromosome condensation	7	6.81×10^{-8}	28.23375	1.09×10^{-4}
BP_FAT	GO:0007093	mitotic cell cycle checkpoint	10	8.99×10^{-8}	12.19398	1.44×10^{-4}
BP_FAT	GO:0006270	DNA replication initiation	7	3.03×10^{-7}	22.93992	4.86×10^{-4}
BP_FAT	GO:0034621	cellular macromolecular complex subunit organization	24	3.30×10^{-7}	3.524982	5.30×10^{-4}
BP_FAT	GO:0031570	DNA integrity checkpoint	10	5.05×10^{-7}	10.08348	8.11×10^{-4}
BP_FAT	GO:0065004	protein-DNA complex assembly	12	1.16×10^{-6}	6.914388	0.001864
BP_FAT	GO:0034622	cellular macromolecular complex assembly	21	2.89×10^{-6}	3.46263	0.004632
BP_FAT	GO:0008283	cell proliferation	25	3.01×10^{-6}	3.006543	0.004837
BP_FAT	GO:0006334	nucleosome assembly	11	4.13×10^{-6}	6.866371	0.006625
BP_FAT	GO:0031497	chromatin assembly	11	5.69×10^{-6}	6.6296	0.009132
BP_FAT	GO:0051656	establishment of organelle localization	10	5.87×10^{-6}	7.599146	0.009425
BP_FAT	GO:0050000	chromosome localization	6	6.12×10^{-6}	20.97364	0.009814
BP_FAT	GO:0007052	mitotic spindle organization	6	6.12×10^{-6}	20.97364	0.009814
BP_FAT	GO:0051303	establishment of chromosome localization	6	6.12×10^{-6}	20.97364	0.009814

BP_FAT	GO:0000079	regulation of cyclin-dependent protein kinase activity	9	7.44×10^{-6}	8.739018	0.01193
BP_FAT	GO:0040001	establishment of mitotic spindle localization	5	8.39×10^{-6}	32.77132	0.013461
BP_FAT	GO:0051640	organelle localization	11	9.44×10^{-6}	6.269296	0.015145
BP_FAT	GO:0051783	regulation of nuclear division	9	9.82×10^{-6}	8.42691	0.015762
BP_FAT	GO:0007088	regulation of mitosis	9	9.82×10^{-6}	8.42691	0.015762
BP_FAT	GO:0034728	nucleosome organization	11	1.04×10^{-5}	6.201884	0.01669
BP_FAT	GO:0000910	cytokinesis	8	1.07×10^{-5}	10.23105	0.017097
BP_FAT	GO:0042770	DNA damage response, signal transduction	10	2.00×10^{-5}	6.554264	0.032156
BP_FAT	GO:0000725	recombinational repair	6	2.23×10^{-5}	16.55814	0.035703
BP_FAT	GO:0000724	double-strand break repair via homologous recombination	6	2.23×10^{-5}	16.55814	0.035703
BP_FAT	GO:0051293	establishment of spindle localization	5	2.44×10^{-5}	26.21705	0.03918
BP_FAT	GO:0051653	spindle localization	5	2.44×10^{-5}	26.21705	0.03918
BP_FAT	GO:0051297	centrosome organization	7	2.65×10^{-5}	11.46996	0.042578
BP_FAT	GO:0000077	DNA damage checkpoint	8	3.12×10^{-5}	8.739018	0.049988
CC_FAT	GO:0005694	chromosome	77	3.37×10^{-56}	10.04505	4.32×10^{-53}
CC_FAT	GO:0044427	chromosomal part	70	4.64×10^{-53}	10.88253	5.95×10^{-50}
CC_FAT	GO:0000793	condensed chromosome	42	4.21×10^{-42}	19.53794	5.40×10^{-39}
CC_FAT	GO:0000775	chromosome, centromeric region	39	2.51×10^{-38}	18.87392	3.22×10^{-35}
CC_FAT	GO:0043232	intracellular non-membrane-bounded organelle	129	8.05×10^{-38}	2.981977	1.03×10^{-34}
CC_FAT	GO:0043228	non-membrane-bounded organelle	129	8.05×10^{-38}	2.981977	1.03×10^{-34}
CC_FAT	GO:0005819	spindle	39	3.52×10^{-35}	15.92086	4.51×10^{-32}
CC_FAT	GO:0015630	microtubule cytoskeleton	60	4.02×10^{-32}	6.558403	5.16×10^{-29}
CC_FAT	GO:0000779	condensed chromosome, centromeric region	28	2.16×10^{-31}	25.45853	2.78×10^{-28}
CC_FAT	GO:0000777	condensed chromosome kinetochore	25	3.84×10^{-28}	25.86612	4.93×10^{-25}
CC_FAT	GO:0005654	nucleoplasm	67	9.86×10^{-27}	4.558536	1.27×10^{-23}
CC_FAT	GO:0031981	nuclear lumen	84	3.89×10^{-26}	3.476406	4.99×10^{-23}
CC_FAT	GO:0000776	kinetochore	26	3.93×10^{-26}	20.26291	5.04×10^{-23}
CC_FAT	GO:0070013	intracellular organelle lumen	90	4.37×10^{-24}	3.035888	5.60×10^{-21}
CC_FAT	GO:0043233	organelle lumen	90	2.20×10^{-23}	2.967497	2.82×10^{-20}
CC_FAT	GO:0031974	membrane-enclosed lumen	90	8.75×10^{-23}	2.909938	1.12×10^{-19}
CC_FAT	GO:0044430	cytoskeletal part	61	2.33×10^{-20}	3.845139	2.98×10^{-17}
CC_FAT	GO:0005874	microtubule	30	1.21×10^{-15}	6.570371	1.57×10^{-12}
CC_FAT	GO:0000228	nuclear chromosome	23	2.43×10^{-14}	8.519852	3.12×10^{-11}
CC_FAT	GO:0005815	microtubule organizing center	27	8.42×10^{-14}	6.404164	1.08×10^{-10}
CC_FAT	GO:0005856	cytoskeleton	63	8.48×10^{-14}	2.737575	1.09×10^{-10}
CC_FAT	GO:0005813	centrosome	24	2.55×10^{-12}	6.429577	3.27×10^{-9}
CC_FAT	GO:0005876	spindle microtubule	11	1.94×10^{-11}	22.76218	2.49×10^{-8}
CC_FAT	GO:0000922	spindle pole	11	1.18×10^{-10}	19.4148	1.51×10^{-7}
CC_FAT	GO:0005657	replication fork	10	1.62×10^{-9}	18.75293	2.07×10^{-6}
CC_FAT	GO:0044454	nuclear chromosome part	15	1.37×10^{-8}	7.378204	1.76×10^{-5}
CC_FAT	GO:0000796	condensin complex	5	1.08×10^{-6}	50.00782	0.001379
CC_FAT	GO:0000940	outer kinetochore of condensed chromosome	5	8.68×10^{-6}	33.33855	0.011141
CC_FAT	GO:0032993	protein-DNA complex	10	1.22×10^{-5}	6.977836	0.015656
CC_FAT	GO:0031262	Ndc80 complex	4	1.78×10^{-5}	60.00939	0.022803
CC_FAT	GO:0000785	chromatin	14	2.88×10^{-5}	4.200657	0.03688
MF_FAT	GO:0005524	ATP binding	63	5.49×10^{-13}	2.624537	7.54×10^{-10}
MF_FAT	GO:0032559	adenyl ribonucleotide binding	63	9.98×10^{-13}	2.589473	1.37×10^{-9}
MF_FAT	GO:0030554	adenyl nucleotide binding	63	9.75×10^{-12}	2.458111	1.34×10^{-8}
MF_FAT	GO:0001883	purine nucleoside binding	63	1.87×10^{-11}	2.421262	2.57×10^{-8}
MF_FAT	GO:0001882	nucleoside binding	63	2.50×10^{-11}	2.40474	3.44×10^{-8}
MF_FAT	GO:0003677	DNA binding	79	2.57×10^{-11}	2.085343	3.54×10^{-8}
MF_FAT	GO:0032555	purine ribonucleotide binding	68	3.01×10^{-11}	2.278919	4.14×10^{-8}
MF_FAT	GO:0032553	ribonucleotide binding	68	3.01×10^{-11}	2.278919	4.14×10^{-8}
MF_FAT	GO:0000166	nucleotide binding	77	3.05×10^{-11}	2.110411	4.19×10^{-8}
MF_FAT	GO:0017076	purine nucleotide binding	68	2.11×10^{-10}	2.181488	2.90×10^{-7}
MF_FAT	GO:0003777	microtubule motor activity	12	4.01×10^{-8}	9.589216	5.51×10^{-5}
MF_FAT	GO:0043566	structure-specific DNA binding	14	6.34×10^{-7}	5.940905	8.72×10^{-4}
MF_FAT	GO:0003697	single-stranded DNA binding	9	2.60×10^{-6}	10.06868	0.003574
MF_FAT	GO:0008094	DNA-dependent ATPase activity	9	3.43×10^{-6}	9.71539	0.004718

MF_FAT	GO:0003887	DNA-directed DNA polymerase activity	7	1.06×10^{-5}	13.45986	0.014623
MF_FAT	GO:0003684	damaged DNA binding	8	1.45×10^{-5}	9.844929	0.019952
MF_FAT	GO:0003774	motor activity	12	1.91×10^{-5}	5.199786	0.026275
MF_FAT	GO:0003682	chromatin binding	12	3.19×10^{-5}	4.922464	0.043838
KEGG_PATHWAY	hsa04110	Cell cycle	31	2.66×10^{-25}	12.12577	2.64×10^{-22}
KEGG_PATHWAY	hsa03030	DNA replication	18	2.56×10^{-20}	24.44712	2.54×10^{-17}
KEGG_PATHWAY	hsa03430	Mismatch repair	11	6.87×10^{-12}	23.3842	6.81×10^{-9}
KEGG_PATHWAY	hsa04114	Oocyte meiosis	15	2.95×10^{-8}	6.667395	2.92×10^{-5}
KEGG_PATHWAY	hsa03420	Nucleotide excision repair	10	1.59×10^{-7}	11.11233	1.57×10^{-4}
KEGG_PATHWAY	hsa03410	Base excision repair	8	4.81×10^{-6}	11.17582	0.004769
KEGG_PATHWAY	hsa04115	p53 signaling pathway	10	7.38×10^{-6}	7.190328	0.007312
KEGG_PATHWAY	hsa03440	Homologous recombination	7	1.57×10^{-5}	12.22356	0.015522
KEGG_PATHWAY	hsa00240	Pyrimidine metabolism	11	1.81×10^{-5}	5.661437	0.017917

Table S3. GSEA (GO gene sets) for TACC3 high expression patients.

Name	Size	ES	NES	NOM *p*-Value	FDR *q*-Value
CELL_CYCLE_PHASE	165	0.70621014	2.4877517	0	0
CELL_CYCLE_PROCESS	185	0.70382446	2.4227076	0	0
M_PHASE_OF_MITOTIC_CELL_CYCLE	84	0.77222216	2.392277	0	0
MITOSIS	81	0.7718224	2.3819587	0	0
REGULATION_OF_MITOSIS	40	0.7665291	2.3412144	0	0
MITOTIC_CELL_CYCLE	151	0.69491	2.322767	0	0
DNA_RECOMBINATION	44	0.70968705	2.3178174	0	0
INTERPHASE_OF_MITOTIC_CELL_CYCLE	62	0.6613634	2.2932644	0	9.61×10^{-5}
CELL_CYCLE_GO_0007049	306	0.6220858	2.2804923	0	8.65×10^{-5}
INTERPHASE	68	0.6482021	2.274392	0	7.86×10^{-5}
DNA_METABOLIC_PROCESS	244	0.6140975	2.2492678	0	1.98×10^{-4}
CONDENSED_CHROMOSOME	32	0.78633547	2.2439635	0	1.83×10^{-4}
CHROMOSOME	121	0.7219317	2.2205505	0	3.33×10^{-4}
CELL_CYCLE_CHECKPOINT_GO_0000075	47	0.8059204	2.2069228	0	3.11×10^{-4}
CHROMOSOMAL_PART	94	0.7289192	2.2059443	0	2.91×10^{-4}
DNA_REPLICATION	93	0.64487696	2.2016928	0	2.74×10^{-4}
MITOTIC_CELL_CYCLE_CHECKPOINT	21	0.8370806	2.1892972	0	3.03×10^{-4}
NUCLEAR_CHROMOSOME	53	0.6495099	2.171287	0	4.61×10^{-4}
SISTER_CHROMATID_SEGREGATION	17	0.8527708	2.1581433	0	6.54×10^{-4}
CHROMOSOME_ORGANIZATION_AND_BIOGENESIS	118	0.60518193	2.146648	0	8.38×10^{-4}
CHROMATIN_BINDING	32	0.7167828	2.1380012	0	8.34×10^{-4}
MITOTIC_SISTER_CHROMATID_SEGREGATION	16	0.86155313	2.1352282	0	8.30×10^{-4}
DNA_PACKAGING	33	0.63601506	2.121751	0.002028398	9.89×10^{-4}
RESPONSE_TO_DNA_DAMAGE_STIMULUS	158	0.60158515	2.1134984	0	9.42×10^{-4}
CHROMOSOME_SEGREGATION	31	0.74191225	2.10383	0	9.54×10^{-4}
CHROMATIN	34	0.6671679	2.0997033	0	0.001004021
REGULATION_OF_CELL_CYCLE	180	0.5700722	2.0977573	0	9.69×10^{-4}
ORGANELLE_LOCALIZATION	23	0.6899464	2.089862	0	0.001024654
G1_PHASE	15	0.73988986	2.0759387	0	0.001220265
REGULATION_OF_DNA_METABOLIC_PROCESS	44	0.62276834	2.0634596	0	0.001571102
RESPONSE_TO_ENDOGENOUS_STIMULUS	195	0.52656674	2.059465	0	0.001722271
DNA_INTEGRITY_CHECKPOINT	23	0.7635856	2.0593152	0	0.001671616
CELL_DIVISION	17	0.77510244	2.0587893	0.001964637	0.001648203
DNA_POLYMERASE_ACTIVITY	18	0.80629694	2.0492055	0	0.001764032
CYTOKINESIS	15	0.8105935	2.0456033	0	0.001861392
DNA_DEPENDENT_DNA_REPLICATION	49	0.6634596	2.0426996	0	0.001832831

ESTABLISHMENT_OF_ORGANELLE_LOCALIZATION	16	0.80583733	2.0332205	0	0.002123206
SPINDLE	38	0.77936935	2.0275178	0	0.002247689
MEIOSIS_I	16	0.74679804	2.0267758	0.00204499	0.002211241
MOTOR_ACTIVITY	28	0.6500422	1.9993869	0.001926782	0.003592489
DNA_DEPENDENT_ATPASE_ACTIVITY	22	0.7588351	1.9868622	0.003944773	0.004227612
REGULATION_OF_CYCLIN_DEPENDENT_PROTEIN_KINASE_ACTIVITY	43	0.6360456	1.9847379	0	0.00425203
DNA_HELICASE_ACTIVITY	25	0.71781224	1.9700005	0.003846154	0.005314991
CONDENSED_NUCLEAR_CHROMOSOME	17	0.7211337	1.9603025	0.006048387	0.005782867
MEIOTIC_CELL_CYCLE	31	0.62992895	1.9552591	0.002083333	0.006075959
SPINDLE_POLE	18	0.76651454	1.9435581	0.001968504	0.006865295
MICROTUBULE_MOTOR_ACTIVITY	16	0.7946937	1.9338679	0	0.007617997
DEOXYRIBONUCLEASE_ACTIVITY	22	0.6138449	1.9275761	0	0.008184224
REGULATION_OF_TRANSCRIPTION_FROM_RNA_POLYMERASE_II_PROMOTER	286	0.4284022	1.9094104	0	0.009995976
MICROTUBULE_ORGANIZING_CENTER_PART	19	0.6753911	1.8999242	0.001915709	0.011107863
DNA_DAMAGE_RESPONSESIGNAL_TRANSDUCTION	35	0.5735531	1.8978816	0	0.011174887
REGULATION_OF_DNA_REPLICATION	19	0.6335172	1.8971134	0.007707129	0.011107754
REGULATION_OF_RNA_METABOLIC_PROCESS	460	0.41384968	1.8893718	0	0.011805994
REGULATION_OF_MITOTIC_CELL_CYCLE	23	0.61966664	1.8815342	0.001901141	0.01276054
REGULATION_OF_TRANSCRIPTIONDNA_DEPENDENT	454	0.41099814	1.8805015	0	0.012674831
DNA_DAMAGE_CHECKPOINT	20	0.6791195	1.8787233	0	0.012740776
CHROMOSOMEPERICENTRIC_REGION	31	0.7954568	1.871907	0.001964637	0.013514848
NUCLEAR_CHROMOSOME_PART	33	0.5777887	1.8695798	0.001923077	0.013589866
ENDONUCLEASE_ACTIVITY	25	0.62093097	1.8611847	0	0.014505335
RNA_POLYMERASE_II_TRANSCRIPTION_FACTOR_ACTIVITY	180	0.42295092	1.8572562	0	0.014951336
REPLICATION_FORK	18	0.788648	1.8551772	0.001865672	0.014986946
ESTABLISHMENT_AND_OR_MAINTENANCE_OF_CHROMATIN_ARCHITECTURE	73	0.5491656	1.8549811	0.005882353	0.014828004
MICROTUBULE_ORGANIZING_CENTER	64	0.55780953	1.8427411	0.003787879	0.016762368
TRANSCRIPTION_FROM_RNA_POLYMERASE_II_PROMOTER	452	0.40887722	1.8358023	0	0.017717859
PROTEIN_N_TERMINUS_BINDING	37	0.5249159	1.8291347	0.005703422	0.018657427
DOUBLE_STRANDED_DNA_BINDING	32	0.6507873	1.8281395	0	0.018640153
NUCLEASE_ACTIVITY	55	0.5127347	1.8239105	0	0.019329093
BASE_EXCISION_REPAIR	16	0.70484656	1.8142556	0.001923077	0.021282393
N_ACETYLTRANSFERASE_ACTIVITY	17	0.639989	1.8140831	0.009578544	0.021021206
NUCLEAR_BODY	30	0.5884752	1.8140578	0.001941748	0.020729244
SEQUENCE_SPECIFIC_DNA_BINDING	53	0.47416455	1.8127787	0.003795066	0.02072364
CENTROSOME	55	0.5694155	1.8084015	0.003868472	0.02148948
REGULATION_OF_GENE_EXPRESSION_EPIGENETIC	29	0.54041135	1.7975414	0.011494253	0.023904964
SPINDLE_MICROTUBULE	15	0.8108091	1.7967873	0.001960784	0.02379903
NUCLEAR_LUMEN	376	0.5115914	1.7963427	0	0.023622751
DOUBLE_STRAND_BREAK_REPAIR	23	0.6339169	1.7961807	0.003752345	0.023346854
KINETOCHORE	25	0.77661735	1.7951962	0.001953125	0.023396878
CYTOSKELETAL_PART	228	0.4105887	1.7918798	0	0.023911811
S_ADENOSYLMETHIONINE_DEPENDENT_METHYLTRANSFERASE_ACTIVITY	23	0.62934077	1.7903534	0.003929273	0.024051256
MICROTUBULE_CYTOSKELETON	147	0.5201849	1.7902784	0	0.023767862
STRUCTURE_SPECIFIC_DNA_BINDING	55	0.58455	1.7822909	0	0.025870707
RESPONSE_TO_ABIOTIC_STIMULUS	87	0.39772093	1.7779504	0.001972387	0.02682391
N_ACYLTRANSFERASE_ACTIVITY	18	0.6102927	1.7768737	0.013333334	0.026701175
METHYLTRANSFERASE_ACTIVITY	35	0.5982532	1.7740124	0.001901141	0.027045373
NUCLEOTIDYLTRANSFERASE_ACTIVITY	47	0.56983835	1.7724351	0	0.027209636
NEGATIVE_REGULATION_OF_DNA_METABOLIC_PROCESS	17	0.6301616	1.7680393	0.009416196	0.028002908
HELICASE_ACTIVITY	50	0.6241985	1.7606634	0.005791506	0.02948965
ORGANELLE_LUMEN	446	0.49884555	1.7595387	0	0.029463269
MEMBRANE_ENCLOSED_LUMEN	446	0.49884555	1.7595387	0	0.029139496
MICROTUBULE_ORGANIZING_CENTER_ORGANIZATION_AND_BIOGENESIS	15	0.67708325	1.7590375	0.007677543	0.028886056

SINGLE_STRANDED_DNA_BINDING	34	0.6339183	1.7572346	0.001831502	0.029250527
ORGANELLE_ORGANIZATION_AND_BIOGENESIS	457	0.39555433	1.7572345	0	0.02893935
POSITIVE_REGULATION_OF_RNA_METABOLIC_PROCESS	118	0.42599612	1.7546059	0	0.029414074
RESPONSE_TO_RADIATION	58	0.4298815	1.752643	0.002061856	0.02965334
POSITIVE_REGULATION_OF_TRANSCRIPTIONDNA_DEPENDENT	116	0.42686847	1.7522273	0	0.029458513
CYTOSKELETON	357	0.353476	1.7441908	0	0.03202136
CHROMATIN_MODIFICATION	51	0.5225477	1.7394614	0.00390625	0.03322551
NUCLEOPLASM	270	0.48371434	1.736006	0	0.033976775
THYROID_HORMONE_RECEPTOR_BINDING	17	0.63357776	1.7325631	0.013565891	0.034748856
G1_S_TRANSITION_OF_MITOTIC_CELL_CYCLE	27	0.56768495	1.7324148	0.008048289	0.03444366
TRANSFERASE_ACTIVITY_TRANSFERRING_ONE_CARBON_GROUPS	36	0.58104545	1.7317364	0.001890359	0.034341037
POSITIVE_REGULATION_OF_NUCLEOBASENUCLEOSIDENUCLEOTIDE_AND_NUCLEIC_ACID_METABOLIC_PROCESS	148	0.39781955	1.7305148	0	0.034304682
ATP_BINDING	152	0.3738572	1.7277309	0	0.035047684
ACETYLTRANSFERASE_ACTIVITY	19	0.6024418	1.7148277	0.021359224	0.039336685
MICROTUBULE	30	0.614468	1.7143352	0.007827789	0.039155103
RNA_PROCESSING	154	0.60661757	1.7114264	0.007766991	0.039804284
POSITIVE_REGULATION_OF_TRANSCRIPTION	138	0.393046	1.7019444	0.001901141	0.043042794
DAMAGED_DNA_BINDING	20	0.67358613	1.7014157	0.003780718	0.042929158
PROTEIN_DNA_COMPLEX_ASSEMBLY	48	0.5417091	1.6928593	0.015355086	0.04589337
ADENYL_RIBONUCLEOTIDE_BINDING	159	0.36933994	1.6904402	0	0.046443332
NEGATIVE_REGULATION_OF_TRANSCRIPTION_DNA_DEPENDENT	128	0.4161575	1.689301	0.019305019	0.046471145
NEGATIVE_REGULATION_OF_RNA_METABOLIC_PROCESS	128	0.4161575	1.689301	0.019305019	0.0460635

NES, Normalized enrichment score, ES, Enrichment Score, NOM, Normalized.

Table S4. GSEA (KEGG) for TACC3 high expression patients.

**Name**	**Size**	**ES**	**NES**	**NOM *p*-Value**	**FDR *q*-Value**
KEGG_HOMOLOGOUS_RECOMBINATION	26	0.80759203	2.213977	0	0
KEGG_BASE_EXCISION_REPAIR	33	0.7432589	2.1963007	0	0
KEGG_CELL_CYCLE	114	0.74472386	2.1878688	0	0
KEGG_SYSTEMIC_LUPUS_ERYTHEMATOSUS	125	0.6783835	2.1286476	0	0.001906133
KEGG_DNA_REPLICATION	36	0.84118605	1.985183	0	0.012693218
KEGG_PYRIMIDINE_METABOLISM	94	0.5864074	1.9687493	0	0.013036668
KEGG_PROGESTERONE_MEDIATED_OOCYTE_MATURATION	83	0.5074202	1.922757	0	0.01847208
KEGG_MISMATCH_REPAIR	23	0.7675387	1.9150902	0.001972387	0.017438423
KEGG_P53_SIGNALING_PATHWAY	66	0.54788786	1.8679833	0	0.027084913
KEGG_NUCLEOTIDE_EXCISION_REPAIR	43	0.6376394	1.8496778	0.002016129	0.029494127
KEGG_BLADDER_CANCER	42	0.526119	1.8167424	0.009765625	0.0374283

Table S5. GSEA (oncogenic signature) for TACC3 high expression patients.

Name	Size	ES	NES	NOM *p*-Value	FDR *q*-Value
PRC2_EZH2_UP.V1_UP	183	0.57203525	2.403676	0	0
RPS14_DN.V1_DN	182	0.61784184	2.2823296	0	0
RB_P130_DN.V1_UP	125	0.5468725	2.2411666	0	0
E2F1_UP.V1_UP	183	0.5724871	2.2111328	0	0
RB_P107_DN.V1_UP	133	0.68314743	2.186367	0	0
PRC2_EDD_UP.V1_UP	189	0.53454906	2.1687953	0	0
HOXA9_DN.V1_DN	185	0.5069483	2.1662169	0	0
CSR_LATE_UP.V1_UP	169	0.64441127	2.159606	0	0
VEGF_A_UP.V1_DN	188	0.5457499	1.9342508	0.001872659	0.00344927
GCNP_SHH_UP_LATE.V1_UP	172	0.535935	1.8970882	0.001953125	0.00473437
GCNP_SHH_UP_EARLY.V1_UP	171	0.49369073	1.8265511	0.001964637	0.00976253
MYC_UP.V1_UP	168	0.41754362	1.8023283	0.008032128	0.01275091
MTOR_UP.V1_UP	162	0.40045226	1.7130936	0.003816794	0.02699401
ESC_J1_UP_LATE.V1_DN	180	0.37744802	1.6929737	0.001897533	0.03016229
CORDENONSI_YAP_CONSERVED_SIGNATURE	57	0.50757056	1.6493119	0.032075472	0.04206474

Table S6. GSEA (Hallmark gene sets) for TACC3 high expression patients.

Name	Size	ES	NES	NOM *p*-Value	FDR *q*-Value
HALLMARK_G2M_CHECKPOINT	191	0.809857	2.181284	0	0.003141
HALLMARK_E2F_TARGETS	190	0.822553	2.046442	0	0.008003
HALLMARK_MYC_TARGETS_V2	57	0.678937	1.942825	0	0.016417
HALLMARK_MITOTIC_SPINDLE	195	0.523238	1.891097	0	0.02143
HALLMARK_DNA_REPAIR	146	0.594399	1.760913	0	0.048998
HALLMARK_INTERFERON_GAMMA_RESPONSE	196	0.159735	0.452687	0.919028	0.972879

Table S7. GSEA (curated gene sets) for TACC3 high expression patients.

Name	Size	ES	NES	NOM *p*-Value	FDR *q*-Value
ABRAMSON_INTERACT_WITH_AIRE	42	0.7928517	2.0061295	0	0.006346698
ACEVEDO_LIVER_CANCER_WITH_H3K9ME3_DN	109	0.52613	2.0733776	0	0.003555315
AFFAR_YY1_TARGETS_DN	227	0.6503626	2.4505792	0	0
ALCALAY_AML_BY_NPM1_LOCALIZATION_DN	182	0.59235257	2.2365808	0.00203252	8.37×10^{-4}
AMUNDSON_GAMMA_RADIATION_RESPONSE	39	0.9159147	2.0648818	0	0.003821157
AUNG_GASTRIC_CANCER	53	0.47144455	1.8035903	0	0.03308335
BASAKI_YBX1_TARGETS_UP	276	0.7048891	2.2058778	0	0.001016619
BENPORATH_ES_1	365	0.5566054	2.0675218	0	0.003739914
BENPORATH_ES_2	35	0.7373229	2.1881607	0	0.001272804
BENPORATH_ES_CORE_NINE_CORRELATED	98	0.6384028	2.1593273	0	0.001609861
BENPORATH_PROLIFERATION	143	0.7832047	1.8205001	0.003960396	0.029353224
BHATI_G2M_ARREST_BY_2METHOXYESTRADIOL_UP	118	0.54629517	1.8488202	0.003831418	0.023196854
BHATTACHARYA_EMBRYONIC_STEM_CELL	86	0.6446279	1.9983622	0	0.006635406
BIDUS_METASTASIS_UP	207	0.6518633	1.8596448	0.003898636	0.021303887
BILD_MYC_ONCOGENIC_SIGNATURE	191	0.470867	1.953322	0.003891051	0.009729113
BLUM_RESPONSE_TO_SALIRASIB_DN	324	0.693627	2.0955734	0	0.003074553
BOHN_PRIMARY_IMMUNODEFICIENCY_SYNDROM_UP	45	0.61907387	1.8140701	0.007736944	0.030778974
BOYAULT_LIVER_CANCER_SUBCLASS_G123_UP	43	0.72793293	1.800927	0.001949318	0.033686325
BOYAULT_LIVER_CANCER_SUBCLASS_G23_UP	51	0.7417551	1.9299381	0.001992032	0.011823437
BOYAULT_LIVER_CANCER_SUBCLASS_G3_UP	187	0.63929415	1.7647958	0.001984127	0.043754566
BOYLAN_MULTIPLE_MYELOMA_C_CLUSTER_UP	37	0.50292206	1.8300333	0.006012024	0.026947046
BROWNE_HCMV_INFECTION_2HR_DN	49	0.49815258	1.7909303	0.005660377	0.036399074
BURTON_ADIPOGENESIS_3	98	0.8441698	2.0486622	0	0.004201578
BURTON_ADIPOGENESIS_PEAK_AT_16HR	40	0.8134809	2.0542796	0	0.004114397

BURTON_ADIPOGENESIS_PEAK_AT_24HR	41	0.8277658	2.111167	0	0.002778943
CAFFAREL_RESPONSE_TO_THC_DN	28	0.70306015	1.8741688	0	0.019191617
CAIRO_PML_TARGETS_BOUND_BY_MYC_UP	23	0.80442816	1.9657879	0	0.008851073
CHANG_CORE_SERUM_RESPONSE_UP	207	0.6249973	1.8672616	0.001992032	0.02003309
CHANG_CYCLING_GENES	141	0.8613869	2.1340013	0	0.002061518
CHAUHAN_RESPONSE_TO_METHOXYESTRADIOL_UP	46	0.5838064	1.7479134	0.006036217	0.049088128
CHEMNITZ_RESPONSE_TO_PROSTAGLANDIN_E2_UP	137	0.7468303	2.2482142	0	0.001011845
CHEN_ETV5_TARGETS_TESTIS	21	0.7311871	1.9357517	0.004008016	0.011510383
CHEN_HOXA5_TARGETS_9HR_DN	40	0.58707553	2.0013084	0	0.006536772
CHIANG_LIVER_CANCER_SUBCLASS_PROLIFERATION_UP	168	0.77120334	2.2239032	0	8.38×10^{-4}
CHIARETTI_T_ALL_RELAPSE_PROGNOSIS	19	0.73424876	1.9358388	0	0.011568487
CHICAS_RB1_TARGETS_GROWING	237	0.61510146	2.138886	0	0.00200637
CHICAS_RB1_TARGETS_LOW_SERUM	87	0.6470734	2.096888	0.001953125	0.003137361
CHIN_BREAST_CANCER_COPY_NUMBER_UP	26	0.62348527	1.9947418	0	0.006849341
COLLER_MYC_TARGETS_UP	24	0.7574644	1.971261	0	0.008360074
COLLIS_PRKDC_SUBSTRATES	19	0.6779687	1.9378507	0.002012072	0.011365364
CONCANNON_APOPTOSIS_BY_EPOXOMICIN_DN	167	0.52107465	2.0136642	0.002028398	0.005980703
CROONQUIST_IL6_DEPRIVATION_DN	96	0.8818544	2.0591085	0	0.003886472
CROONQUIST_NRAS_SIGNALING_DN	71	0.9129002	2.016758	0	0.005895814
CROONQUIST_NRAS_VS_STROMAL_STIMULATION_DN	96	0.72205734	2.3096159	0	7.97×10^{-4}
CUI_TCF21_TARGETS_2_UP	411	0.5261993	2.237032	0	8.70×10^{-4}
DELPUECH_FOXO3_TARGETS_DN	39	0.75366247	1.9862095	0	0.007182398
DEURIG_T_CELL_PROLYMPHOCYTIC_LEUKEMIA_UP	349	0.38681054	1.8945023	0	0.016231429
DOANE_RESPONSE_TO_ANDROGEN_DN	230	0.38159314	1.7498947	0	0.04839371
DORMOY_ELAVL1_TARGETS	16	0.6298957	1.8052552	0.003824092	0.032813106
DUTERTRE_ESTRADIOL_RESPONSE_24HR_UP	310	0.81336683	2.335335	0	8.17×10^{-4}
DUTERTRE_ESTRADIOL_RESPONSE_6HR_UP	222	0.49134362	1.9869558	0	0.007252167
FAELT_B_CLL_WITH_VH3_21_UP	42	0.57425183	1.7794455	0.01417004	0.039771494
FARMER_BREAST_CANCER_CLUSTER_2	33	0.9261263	1.9109015	0	0.014334178
FERRANDO_HOX11_NEIGHBORS	23	0.7253846	2.0642395	0.001949318	0.003752396
FERRANDO_T_ALL_WITH_MLL_ENL_FUSION_DN	84	0.66864717	2.0504076	0	0.004220348
FERREIRA_EWINGS_SARCOMA_UNSTABLE_VS_STABLE_UP	160	0.75564826	2.1832142	0	0.001341325
FLECHNER_BIOPSY_KIDNEY_TRANSPLANT_OK_VS_DONOR_DN	24	0.66978097	2.064287	0	0.003795037
FOURNIER_ACINAR_DEVELOPMENT_LATE_2	266	0.6893372	2.0481715	0	0.004175642
FOURNIER_ACINAR_DEVELOPMENT_LATE_DN	21	0.8528276	1.9617832	0.003883495	0.009055816
FRASOR_RESPONSE_TO_SERM_OR_FULVESTRANT_DN	49	0.86990434	2.0799599	0	0.003406358
FUJII_YBX1_TARGETS_DN	202	0.75835854	2.2856722	0	8.56×10^{-4}
FURUKAWA_DUSP6_TARGETS_PCI35_DN	70	0.8226764	2.2701688	0	8.72×10^{-4}
GAJATE_RESPONSE_TO_TRABECTEDIN_DN	17	0.6908039	1.7593706	0.008048289	0.04523529
GAL_LEUKEMIC_STEM_CELL_DN	229	0.55828035	1.945598	0.001912046	0.010621281
GARCIA_TARGETS_OF_FLI1_AND_DAX1_DN	170	0.6136885	2.0893536	0	0.003131174
GARGALOVIC_RESPONSE_TO_OXIDIZED_PHOSPHOLIPIDS_TURQUOISE_DN	51	0.783331	2.1909318	0	0.001272349
GAVIN_FOXP3_TARGETS_CLUSTER_P6	85	0.86823815	2.4903016	0	0
GENTLES_LEUKEMIC_STEM_CELL_DN	18	0.7885199	2.050354	0	0.004192293
GEORGES_CELL_CYCLE_MIR192_TARGETS	59	0.68415016	1.8082368	0	0.03196272

GINESTIER_BREAST_CANCER_20Q13_AMPLIFICATION_DN	151	0.46511516	1.7593974	0.014522822	0.04541743
GINESTIER_BREAST_CANCER_ZNF217_AMPLIFIED_DN	308	0.4712647	1.86787	0.003891051	0.020014552
GOLDRATH_ANTIGEN_RESPONSE	338	0.609529	1.9936316	0.001941748	0.006811249
GRAHAM_CML_DIVIDING_VS_NORMAL_QUIESCENT_UP	177	0.7782986	2.219608	0	8.06×10^{-4}
GRAHAM_CML_QUIESCENT_VS_NORMAL_QUIESCENT_UP	85	0.66403514	2.201823	0	0.001130455
GRAHAM_NORMAL_QUIESCENT_VS_NORMAL_DIVIDING_DN	85	0.84884435	2.0721562	0.001976285	0.003545244
GREENBAUM_E2A_TARGETS_UP	33	0.852928	1.9793087	0.001964637	0.007739089
GROSS_HYPOXIA_VIA_ELK3_AND_HIF1A_DN	95	0.4587004	1.7752728	0.007751938	0.0410606
GROSS_HYPOXIA_VIA_ELK3_ONLY_DN	44	0.5593625	1.9272964	0.003937008	0.012144187
GROSS_HYPOXIA_VIA_ELK3_UP	199	0.48730752	1.821653	0.002020202	0.029233193
HEIDENBLAD_AMPLICON_8Q24_UP	37	0.5352065	1.8013388	0.008032128	0.033712838
HERNANDEZ_MITOTIC_ARREST_BY_DOCETAXEL_1_DN	36	0.68936193	2.0655956	0	0.003808233
HESS_TARGETS_OF_HOXA9_AND_MEIS1_UP	63	0.6242773	1.9348433	0	0.011549884
HOFFMANN_LARGE_TO_SMALL_PRE_BII_LYMPHOCYTE_UP	158	0.79320997	2.1853247	0	0.001333135
HOFFMANN_SMALL_PRE_BII_TO_IMMATURE_B_LYMPHOCYTE_DN	50	0.48316655	1.7788247	0.001968504	0.039807633
HONRADO_BREAST_CANCER_BRCA1_VS_BRCA2	18	0.77472514	2.007847	0	0.006312263
HORIUCHI_WTAP_TARGETS_DN	294	0.69904584	2.0894296	0	0.003175275
HU_GENOTOXIC_DAMAGE_4HR	33	0.81642354	1.999197	0	0.006638297
ISHIDA_E2F_TARGETS	52	0.8831183	1.9535505	0.001972387	0.009858025
JAEGER_METASTASIS_UP	41	0.7302451	2.163269	0	0.001617846
JEON_SMAD6_TARGETS_DN	17	0.7722918	2.061096	0	0.003829333
JOHANSSON_GLIOMAGENESIS_BY_PDGFB_UP	54	0.69036865	1.774243	0.01002004	0.041262448
KAMMINGA_EZH2_TARGETS	41	0.88034284	1.8985591	0	0.015875509
KAMMINGA_SENESCENCE	39	0.5611523	1.9637505	0	0.008998144
KANG_DOXORUBICIN_RESISTANCE_UP	53	0.9092697	1.8830223	0	0.01777016
KANNAN_TP53_TARGETS_DN	21	0.62160134	1.9300708	0	0.011881267
KATSANOU_ELAVL1_TARGETS_DN	143	0.42494392	1.8579258	0.001919386	0.021386469
KAUFFMANN_DNA_REPAIR_GENES	218	0.70366603	2.2196472	0	8.32×10^{-4}
KAUFFMANN_DNA_REPLICATION_GENES	136	0.70416105	2.3120732	0	9.30×10^{-4}
KAUFFMANN_MELANOMA_RELAPSE_UP	56	0.8744849	1.8731551	0	0.019231327
KENNY_CTNNB1_TARGETS_UP	48	0.5693903	1.8409873	0.001901141	0.024569502
KIM_TIAL1_TARGETS	32	0.65733284	1.8468391	0.003937008	0.023404134
KOBAYASHI_EGFR_SIGNALING_24HR_DN	242	0.83674085	2.195011	0	0.00121801
KOINUMA_COLON_CANCER_MSI_UP	16	0.67206407	1.786187	0.009708738	0.037704594
KOKKINAKIS_METHIONINE_DEPRIVATION_48HR_DN	64	0.56692076	1.9602977	0	0.009102018
KOKKINAKIS_METHIONINE_DEPRIVATION_96HR_DN	72	0.63154536	2.1299608	0	0.002170943
KONG_E2F3_TARGETS	96	0.8854851	2.0995445	0	0.00313758
KORKOLA_TERATOMA	38	0.7234994	2.1769705	0	0.001332056
KRASNOSELSKAYA_ILF3_TARGETS_DN	44	0.49319908	1.8786483	0.003937008	0.018531825
LABBE_WNT3A_TARGETS_UP	110	0.52218604	1.9605699	0	0.009137171
LASTOWSKA_NEUROBLASTOMA_COPY_NUMBER_UP	169	0.5615531	1.9847602	0	0.007273667
LE_EGR2_TARGETS_UP	106	0.7874969	2.1320243	0	0.002148539
LE_NEURONAL_DIFFERENTIATION_DN	19	0.86861956	2.1020236	0	0.003045154
LEE_EARLY_T_LYMPHOCYTE_UP	96	0.8886066	2.24141	0	9.12×10^{-4}

LEE_TARGETS_OF_PTCH1_AND_SUFU_UP	53	0.5842675	1.9699066	0	0.008425347
LI_WILMS_TUMOR_ANAPLASTIC_UP	18	0.9043869	1.9070222	0.001968504	0.014650725
LI_WILMS_TUMOR_VS_FETAL_KIDNEY_1_DN	160	0.7236378	2.050453	0	0.004264772
LI_WILMS_TUMOR_VS_FETAL_KIDNEY_2_UP	29	0.58233905	1.7933033	0.009861933	0.035864163
LIN_MELANOMA_COPY_NUMBER_UP	68	0.50917727	1.8360028	0.002070393	0.02566664
LINDGREN_BLADDER_CANCER_CLUSTER_1_DN	365	0.6130705	2.3016338	0	7.44×10^{-4}
LINDGREN_BLADDER_CANCER_CLUSTER_3_UP	314	0.6688243	2.0869682	0	0.003250871
LIU_COMMON_CANCER_GENES	70	0.47925344	1.7469573	0.003898636	0.049269278
LU_TUMOR_ANGIOGENESIS_UP	25	0.630114	1.7683024	0.013461539	0.04254563
LY_AGING_MIDDLE_DN	16	0.91117907	1.7640934	0.007984032	0.043804314
LY_AGING_OLD_DN	55	0.8179595	1.9682822	0	0.008526513
LY_AGING_PREMATURE_DN	29	0.8330318	2.0848722	0	0.003243131
MANALO_HYPOXIA_DN	277	0.72162837	2.0244968	0	0.005693933
MARKEY_RB1_ACUTE_LOF_DN	219	0.7292346	2.2311015	0	8.74×10^{-4}
MARKEY_RB1_CHRONIC_LOF_UP	113	0.55115676	2.0853045	0	0.003278248
MARKS_HDAC_TARGETS_DN	15	0.66744816	1.7948692	0.007984032	0.035478618
MATTIOLI_MGUS_VS_PCL	99	0.63527507	1.9653313	0	0.008906441
MATZUK_MEIOTIC_AND_DNA_REPAIR	38	0.6021915	2.1157348	0	0.00260257
MATZUK_SPERMATOCYTE	70	0.53030306	2.0921814	0	0.003197597
MISSIAGLIA_REGULATED_BY_METHYLATION_DN	115	0.8140735	2.0983703	0	0.003099477
MITSIADES_RESPONSE_TO_APLIDIN_DN	241	0.68894696	2.020264	0	0.005770229
MOLENAAR_TARGETS_OF_CCND1_AND_CDK4_DN	56	0.87200844	1.9724717	0	0.008310368
MORI_EMU_MYC_LYMPHOMA_BY_ONSET_TIME_UP	101	0.6576743	2.0408642	0	0.004530591
MORI_IMMATURE_B_LYMPHOCYTE_DN	87	0.8538868	2.0466597	0	0.004219402
MORI_LARGE_PRE_BII_LYMPHOCYTE_UP	84	0.8437459	2.0204344	0	0.005823657
MORI_MATURE_B_LYMPHOCYTE_DN	74	0.6670731	1.9634746	0	0.008944215
MORI_PRE_BI_LYMPHOCYTE_UP	78	0.80288	2.1940079	0	0.001208688
MUELLER_PLURINET	281	0.66925454	2.1104243	0	0.002753732
NADERI_BREAST_CANCER_PROGNOSIS_UP	49	0.7925154	2.140984	0.001996008	0.002014818
NAKAMURA_CANCER_MICROENVIRONMENT_DN	46	0.72506344	1.8690518	0.002028398	0.019858437
NAKAYAMA_SOFT_TISSUE_TUMORS_PCA2_UP	86	0.7663706	2.3328366	0	6.36×10^{-4}
NUNODA_RESPONSE_TO_DASATINIB_IMATINIB_UP	28	0.71301454	2.038028	0	0.004652562
ODONNELL_TARGETS_OF_MYC_AND_TFRC_DN	44	0.81699497	2.0282528	0.001964637	0.005440815
ODONNELL_TFRC_TARGETS_DN	130	0.7824776	2.4049652	0	3.74×10^{-4}
OLSSON_E2F3_TARGETS_DN	46	0.7289135	2.1686661	0	0.001581677
OXFORD_RALA_OR_RALB_TARGETS_UP	47	0.8410578	2.0780203	0	0.00338271
PAL_PRMT5_TARGETS_UP	195	0.6284724	1.8432491	0	0.024140881
PEART_HDAC_PROLIFERATION_CLUSTER_DN	74	0.68423486	2.3012471	0	6.98×10^{-4}
PETROVA_ENDOTHELIUM_LYMPHATIC_VS_BLOOD_UP	128	0.5616426	1.9933244	0.001964637	0.006784279
PETROVA_PROX1_TARGETS_UP	27	0.6814952	2.0457022	0	0.004191389
PIONTEK_PKD1_TARGETS_DN	18	0.7081107	2.082291	0	0.003323443
POOLA_INVASIVE_BREAST_CANCER_UP	275	0.5482111	1.7448773	0.024667932	0.049995366
PRAMOONJAGO_SOX4_TARGETS_DN	51	0.5747535	1.7538409	0.006012024	0.047015008
PUIFFE_INVASION_INHIBITED_BY_ASCITES_UP	79	0.54861045	1.8587093	0	0.02132133
PUJANA_BRCA_CENTERED_NETWORK	113	0.7953322	1.9782276	0	0.007856787
PUJANA_BRCA2_PCC_NETWORK	408	0.73442763	2.1744046	0	0.001453308
PUJANA_BREAST_CANCER_LIT_INT_NETWORK	100	0.69914764	2.1063063	0	0.00288441

PUJANA_BREAST_CANCER_WITH_BRCA1_MUTATED_UP	52	0.8217164	1.9322517	0	0.011782094
PUJANA_XPRSS_INT_NETWORK	159	0.78755504	2.0479925	0	0.004149194
PYEON_CANCER_HEAD_AND_NECK_VS_CERVICAL_UP	177	0.5853121	1.8922498	0.009784736	0.016529063
PYEON_HPV_POSITIVE_TUMORS_UP	84	0.690731	2.1239674	0.002012072	0.002461689
RAY_TUMORIGENESIS_BY_ERBB2_CDC25A_UP	100	0.45132107	1.8638422	0.005747126	0.020695716
REICHERT_MITOSIS_LIN9_TARGETS	27	0.9107333	1.8868275	0	0.017262464
REN_BOUND_BY_E2F	58	0.86302817	1.9736489	0	0.008229044
RHODES_CANCER_META_SIGNATURE	62	0.73791367	1.874909	0	0.019108308
RHODES_UNDIFFERENTIATED_CANCER	67	0.8396568	1.8397593	0	0.024778571
RIZ_ERYTHROID_DIFFERENTIATION	77	0.6757176	2.2407634	0	8.76×10^{-4}
RIZ_ERYTHROID_DIFFERENTIATION_CCNE1	39	0.5335631	1.8890774	0	0.016931713
RIZ_ERYTHROID_DIFFERENTIATION_HBZ	41	0.57720304	1.9941412	0	0.006825913
ROSTY_CERVICAL_CANCER_PROLIFERATION_CLUSTER	139	0.90458685	2.0080585	0	0.006354115
ROYLANCE_BREAST_CANCER_16Q_COPY_NUMBER_UP	56	0.504762	1.8119991	0.014522822	0.031035814
RUIZ_TNC_TARGETS_DN	136	0.7779997	2.2418475	0	9.52×10^{-4}
SANSOM_APC_MYC_TARGETS	212	0.48403543	1.8739344	0	0.019128805
SANSOM_APC_TARGETS	200	0.41625887	1.8250672	0	0.02835242
SANSOM_APC_TARGETS_REQUIRE_MYC	201	0.61597043	2.1177995	0	0.00259202
SANSOM_APC_TARGETS_UP	121	0.49128205	1.900245	0	0.015600773
SANSOM_WNT_PATHWAY_REQUIRE_MYC	57	0.55196565	1.9095072	0	0.014407007
SARRIO_EPITHELIAL_MESENCHYMAL_TRANSITION_UP	171	0.76504576	2.2455642	0	9.95×10^{-4}
SASAKI_ADULT_T_CELL_LEUKEMIA	173	0.5652278	1.8592678	0	0.021260481
SCHLOSSER_MYC_AND_SERUM_RESPONSE_SYNERGY	31	0.6558275	1.7545944	0.01178782	0.046867296
SCIAN_CELL_CYCLE_TARGETS_OF_TP53_AND_TP73_DN	22	0.8885886	1.9106853	0	0.014284845
SCIBETTA_KDM5B_TARGETS_DN	76	0.59180635	1.7895917	0.004065041	0.036621753
SENGUPTA_NASOPHARYNGEAL_CARCINOMA_UP	278	0.6121283	1.9419894	0.001941748	0.010963162
SERVITJA_LIVER_HNF1A_TARGETS_UP	133	0.512339	2.063592	0	0.003733113
SHEDDEN_LUNG_CANCER_POOR_SURVIVAL_A6	445	0.72763187	2.2005491	0	0.00112123
SHEPARD_BMYB_MORPHOLINO_DN	188	0.5983017	2.4239304	0	6.24×10^{-4}
SHEPARD_BMYB_TARGETS	69	0.7477624	2.3226473	0	9.53×10^{-4}
SHEPARD_CRUSH_AND_BURN_MUTANT_DN	177	0.5942819	2.2270596	0	8.67×10^{-4}
SHIN_B_CELL_LYMPHOMA_CLUSTER_8	36	0.58162844	1.8036369	0.009708738	0.03322564
SHIPP_DLBCL_VS_FOLLICULAR_LYMPHOMA_UP	45	0.7383125	1.8844632	0	0.01757642
SIMBULAN_PARP1_TARGETS_DN	17	0.8369431	1.9338567	0	0.011621799
SLEBOS_HEAD_AND_NECK_CANCER_WITH_HPV_UP	77	0.5869057	1.808843	0.015873017	0.031918976
SMID_BREAST_CANCER_LUMINAL_A_DN	17	0.87340933	2.0065262	0	0.00638163
SMID_BREAST_CANCER_RELAPSE_IN_BRAIN_UP	39	0.5806662	2.0401003	0	0.004556977
SMIRNOV_RESPONSE_TO_IR_6HR_DN	113	0.5849216	2.169968	0	0.001573328
SONG_TARGETS_OF_IE86_CMV_PROTEIN	58	0.8470402	1.9753613	0	0.008096383
SOTIRIOU_BREAST_CANCER_GRADE_1_VS_3_UP	147	0.880693	1.9866827	0	0.007210405
STEIN_ESR1_TARGETS	85	0.5933328	2.1302195	0	0.002188776
STEIN_ESRRA_TARGETS_RESPONSIVE_TO_ESTROGEN_DN	41	0.7676344	2.096009	0.003929273	0.003110878
SU_TESTIS	74	0.6540217	2.178969	0	0.001345624
TANG_SENESCENCE_TP53_TARGETS_DN	55	0.8702906	2.1565464	0	0.001626642
TARTE_PLASMA_CELL_VS_PLASMABLAST_DN	303	0.6183744	1.7705542	0.004056795	0.042074446

THILLAINADESAN_ZNF217_TARGETS_UP	42	0.6973599	1.9488354	0	0.010284202
TOYOTA_TARGETS_OF_MIR34B_AND_MIR34C	422	0.6050601	2.1597004	0	0.001629961
VANTVEER_BREAST_CANCER_ESR1_DN	232	0.52506524	1.9077644	0.001890359	0.014600384
VANTVEER_BREAST_CANCER_METASTASIS_DN	116	0.6832721	1.8974277	0	0.015920373
VANTVEER_BREAST_CANCER_POOR_PROGNOSIS	51	0.569991	1.9549475	0.001930502	0.009766687
VECCHI_GASTRIC_CANCER_EARLY_UP	403	0.69945294	2.4216504	0	4.68×10^{-4}
VERNELL_RETINOBLASTOMA_PATHWAY_UP	69	0.7961524	2.0150087	0	0.005938648
WAKASUGI_HAVE_ZNF143_BINDING_SITES	57	0.70788443	2.0762348	0	0.003456017
WALLACE_PROSTATE_CANCER_UP	20	0.59808385	1.8159417	0.0078125	0.030489657
WANG_CISPLATIN_RESPONSE_AND_XPC_UP	195	0.56387144	2.3097787	0	8.59×10^{-4}
WANG_METASTASIS_OF_BREAST_CANCER_ESR1_UP	22	0.8354004	1.9534599	0.001972387	0.009790964
WANG_RESPONSE_TO_GSK3_INHIBITOR_SB216763_DN	344	0.66933	2.2633085	0	9.17×10^{-4}
WEIGEL_OXIDATIVE_STRESS_RESPONSE	34	0.5637235	1.7575947	0.004048583	0.04577642
WELCSH_BRCA1_TARGETS_DN	137	0.5633631	1.8146374	0.006048387	0.030720789
WEST_ADRENOCORTICAL_TUMOR_MARKERS_UP	20	0.9098309	2.4012635	0	3.12×10^{-4}
WEST_ADRENOCORTICAL_TUMOR_UP	282	0.60170823	1.7733738	0.002028398	0.04139529
WHITEFORD_PEDIATRIC_CANCER_MARKERS	114	0.88022494	2.0901785	0.001972387	0.003206448
WHITFIELD_CELL_CYCLE_G1_S	135	0.61825395	2.0703034	0.001941748	0.003607133
WHITFIELD_CELL_CYCLE_G2	172	0.69447094	2.32491	0	0.001048391
WHITFIELD_CELL_CYCLE_G2_M	208	0.67309034	2.3330092	0	7.15×10^{-4}
WHITFIELD_CELL_CYCLE_LITERATURE	42	0.892159	1.8505511	0	0.022848621
WHITFIELD_CELL_CYCLE_S	150	0.6350672	2.079723	0	0.003363239
WILCOX_RESPONSE_TO_PROGESTERONE_UP	146	0.5607316	1.9271984	0	0.012084949
WILLIAMS_ESR1_TARGETS_UP	26	0.6552583	2.0815003	0.003898636	0.003352978
WINNEPENNINCKX_MELANOMA_METASTASIS_UP	157	0.8008679	1.9311018	0.001988072	0.011826783
WINTER_HYPOXIA_UP	86	0.5472406	1.7725749	0.015717093	0.041517783
WONG_EMBRYONIC_STEM_CELL_CORE	329	0.7059396	1.8731514	0	0.019129034
WU_APOPTOSIS_BY_CDKN1A_VIA_TP53	52	0.86467487	1.938263	0	0.011403644
XU_CREBBP_TARGETS_UP	25	0.7034419	1.9715642	0	0.008339347
XU_HGF_SIGNALING_NOT_VIA_AKT1_48HR_DN	18	0.7542207	1.8127598	0.001926782	0.030979762
XU_HGF_TARGETS_INDUCED_BY_AKT1_48HR_DN	25	0.7048811	1.8960524	0.003898636	0.0160193
YAMAZAKI_TCEB3_TARGETS_DN	204	0.47713536	1.7850261	0	0.037977442
YANG_BCL3_TARGETS_UP	352	0.40121236	1.830278	0	0.02698069
YAO_TEMPORAL_RESPONSE_TO_PROGESTERONE_CLUSTER_15	33	0.5284339	1.8636894	0.004032258	0.020612208
YU_BAP1_TARGETS	26	0.6942317	1.8470136	0.005859375	0.023493035
YU_MYC_TARGETS_UP	41	0.855911	1.7954935	0	0.035411455
ZHAN_MULTIPLE_MYELOMA_PR_UP	41	0.95540327	1.9449095	0	0.010625209
ZHANG_TLX_TARGETS_36HR_DN	175	0.7203025	1.9185945	0.003976143	0.013165029
ZHANG_TLX_TARGETS_60HR_DN	262	0.77351904	2.0556993	0	0.004057811
ZHANG_TLX_TARGETS_DN	84	0.8203856	1.9177922	0	0.013214158
ZHENG_GLIOBLASTOMA_PLASTICITY_UP	246	0.62638694	2.286519	0	9.07×10^{-4}
ZHOU_CELL_CYCLE_GENES_IN_IR_RESPONSE_24HR	124	0.86601967	2.0043602	0	0.006409275
ZHOU_CELL_CYCLE_GENES_IN_IR_RESPONSE_6HR	84	0.88480496	1.9192615	0	0.013126444
ZWANG_DOWN_BY_2ND_EGF_PULSE	229	0.43577114	1.768852	0.019723866	0.04250819

Table S8. Multivariate analysis of OS-related characteristics with radio- or chemo- therapy.

Variable	Radiotherapy			Chemotherapy		
	p	HR	95% CI	*p*	HR	95% CI
Age (<45 vs. ≥45)	0.682	0.897	0.532–1.511	0.378	0.739	0.378–1.447
WHO (LGG vs. HGG)	0.001	0.179	0.063–0.508	0.073	0.301	0.081–1.117
KPS (≥80 vs. <80)	0	0.302	0.172–0.533	0.029	0.442	0.213–0.919
TACC3 (Low vs. High)	0.006	0.356	0.169–0.748	0.022	0.394	0.178–0.873
IDH1 (Mut vs. WT)	0.368	0.731	0.369–1.446	0.958	0.979	0.448–2.139
